# Comprehensive Statistical and Bioinformatics Analysis in the Deciphering of Putative Mechanisms by Which Lipid-Associated GWAS Loci Contribute to Coronary Artery Disease

**DOI:** 10.3390/biomedicines10020259

**Published:** 2022-01-25

**Authors:** Victor Lazarenko, Mikhail Churilin, Iuliia Azarova, Elena Klyosova, Marina Bykanova, Natalia Ob’edkova, Mikhail Churnosov, Olga Bushueva, Galina Mal, Sergey Povetkin, Stanislav Kononov, Yulia Luneva, Sergey Zhabin, Anna Polonikova, Alina Gavrilenko, Igor Saraev, Maria Solodilova, Alexey Polonikov

**Affiliations:** 1 Department of Surgical Diseases, Institute of Continuing Education, Kursk State Medical University, 3 Karl Marx Street, 305041 Kursk, Russia; lazarenkova@kursksmu.net; 2 Department of Infectious Diseases and Epidemiology, Kursk State Medical University, 3 Karl Marx Street, 305041 Kursk, Russia; gavrilenkoalina2002@yandex.ru; 3 Department of Biological Chemistry, Kursk State Medical University, 3 Karl Marx Street, 305041 Kursk, Russia; azzzzar@yandex.ru; 4 Laboratory of Biochemical Genetics and Metabolomics, Research Institute for Genetic and Molecular Epidemiology, Kursk State Medical University, 18 Yamskaya Street, 305041 Kursk, Russia; ecless@yandex.ru; 5 Laboratory of Genomic Research, Research Institute for Genetic and Molecular Epidemiology, Kursk State Medical University, 18 Yamskaya Street, 305041 Kursk, Russia; marina.bickanova@yandex.ru (M.B.); olga.bushueva@inbox.ru (O.B.); 6 Department of Polyclinical Therapy and General Medical Practice, Kursk State Medical University, 3 Karl Marx Street, 305041 Kursk, Russia; obedkovany@kursksmu.net; 7 Department of Medical Biological Disciplines, Belgorod State University, 85 Pobedy Street, 308015 Belgorod, Russia; churnosov@bsu.edu.ru; 8 Department of Biology, Medical Genetics and Ecology, Kursk State Medical University, 3 Karl Marx Street, 305041 Kursk, Russia; anna-polonikova@rambler.ru (A.P.); solodilovama@kursksmu.net (M.S.); polonikov@rambler.ru (A.P.); 9 Department of Pharmacology, Kursk State Medical University, 3 Karl Marx Street, 305041 Kursk, Russia; malgs@kursksmu.net; 10 Department of Clinical Pharmacology, Kursk State Medical University, 3 Karl Marx Street, 305041 Kursk, Russia; povetkinsv@kursksmu.net (S.P.); lunevajuv@kursksmu.net (Y.L.); 11 Department of Internal Medicine No 2, Kursk State Medical University, 3 Karl Marx Street, 305041 Kursk, Russia; ck325@yandex.ru (S.K.); saraevia@kursksmu.net (I.S.); 12 Department of Surgical Diseases No 1, Kursk State Medical University, 3 Karl Marx Street, 305041 Kursk, Russia; zhabinsn@kursksmu.net; 13 Laboratory of Statistical Genetics and Bioinformatics, Research Institute for Genetic and Molecular Epidemiology, Kursk State Medical University, 18 Yamskaya Street, 305041 Kursk, Russia

**Keywords:** genome-wide association study, plasma lipids, carotid intima-media thickness, coronary artery disease, disease susceptibility

## Abstract

The study was designed to evaluate putative mechanisms by which lipid-associated loci identified by genome-wide association studies (GWAS) are involved in the molecular pathogenesis of coronary artery disease (CAD) using a comprehensive statistical and bioinformatics analysis. A total of 1700 unrelated individuals of Slavic origin from the Central Russia, including 991 CAD patients and 709 healthy controls were examined. Sixteen lipid-associated GWAS loci were selected from European studies and genotyped using the MassArray-4 system. The polymorphisms were associated with plasma lipids such as total cholesterol (rs12328675, rs4846914, rs55730499, and rs838880), LDL-cholesterol (rs3764261, rs55730499, rs1689800, and rs838880), HDL-cholesterol (rs3764261) as well as carotid intima-media thickness/CIMT (rs12328675, rs11220463, and rs1689800). Polymorphisms such as rs4420638 of *APOC1* (*p* = 0.009), rs55730499 of *LPA* (*p* = 0.0007), rs3136441 of *F2* (*p* < 0.0001), and rs6065906 of *PLTP* (*p* = 0.002) showed significant associations with the risk of CAD, regardless of sex, age, and body mass index. A majority of the observed associations were successfully replicated in large independent cohorts. Bioinformatics analysis allowed establishing (1) phenotype-specific and shared epistatic gene–gene and gene–smoking interactions contributing to all studied cardiovascular phenotypes; (2) lipid-associated GWAS loci might be allele-specific binding sites for transcription factors from gene regulatory networks controlling multifaceted molecular mechanisms of atherosclerosis.

## 1. Introduction

Cardiovascular diseases (CVD) were defined by the World Health Organization as the leading cause of death worldwide with an estimated 17.5 million of these cases occurring in 2017, and 42.3% of them were attributed to coronary artery disease (CAD) [1]. CAD is one of the most common cardiovascular disorders responsible for, in addition to high mortality rates, an increased disability as well as a decreased quality of life in the world [1,2]. It is well known that atherosclerosis of coronary arteries is the major cause of CAD, a pathological process affecting elastic and muscular-elastic type of arteries. Disorders of lipid metabolism are characterized by the deposition of cholesterol and atherogenic lipoproteins in the vascular wall, thereby contributing to the development of coronary atherosclerosis [3]. Epidemiological studies have shown that the severity of CAD correlates closely with the levels of plasma cholesterol and low-density lipoproteins [4,5]. Carotid intima-media thickness (CIMT) along with atherogenic lipid metabolism disorders represents well-characterized diagnostic markers for assessing the initial stages of vascular wall modifications underlying atherosclerosis [6].

Coronary artery disease is a typical multifactorial disorder determined by complex interactions between genetic and environmental factors [7]. A wide variety of genomic regions linked to CAD susceptibility has been identified; however, polymorphic genes involved in the regulation of lipid metabolism have attracted greater attention [8,9]. In recent years, genome-wide association studies (GWAS) have discovered a wide range of single nucleotide polymorphisms (SNP) associated with coronary artery disease in various populations of the world, and these data have been deposited into the GWAS catalogue [10]. In particular, several large-scale GWAS have been done and over 15,000 SNP-CAD associations have been identified in 2015, and hundreds of these loci showed substantial effects on the metabolism of lipids and lipoproteins [11,12].

Despite considerable progress in GWAS towards discovering dozens of gene polymorphisms associated with lipid traits, the molecular mechanisms underlying these relationships in the context of CAD pathogenesis remain understudied. The problem is often is exacerbated by the non-replication of associations between GWAS loci and cardiovascular phenotypes in independent populations, which might be attributed to both ethnic specific genetic background and environmental factors [13,14,15]. It is important to note that polymorphic loci associated with lipids are predominantly located in intronic or intergenic regions, thereby complicating pathophysiological interpretation of SNP-phenotype correlation and limiting the clinical use of such markers. In addition, a majority of lipid-associated GWAS loci have not been annotated for their functional role for CAD pathogenesis [9,16], neither by the use of bioinformatics SNP annotation tools nor by assessing with intermediate cardiovascular phenotypes such as carotid intima-media thickness. In this regard, there is a need for comprehensive investigation that, on the one hand, integrates cardiovascular phenotypes such as plasma lipids, CIMT, and CAD, on the other hand, uses modern bioinformatics approaches to deciphering the molecular mechanisms by which lipid-associated GWAS loci are linked to coronary atherosclerosis. Hence, the present study was designed to evaluate putative mechanisms by which lipid-associated GWAS loci are involved in the molecular pathogenesis of coronary artery disease using a comprehensive statistical and bioinformatics analysis.

## 2. Materials and Methods

### 2.1. Study Participants

The study protocol was approved by the Regional Ethics Committee of Kursk State Medical University. All participants gave their informed consent for participating in this study. DNA samples were obtained from the biobank of Research Institute for Genetic and Molecular Epidemiology of Kursk State Medical University. The samples were collected previously in the frames of genetic studies of cardiovascular and other chronic diseases for the period from 2003 to 2018 [17,18,19,20,21,22,23]. A total number of participants comprises 1700 unrelated individuals of Slavic origin, inhabitants of the Kursk region, and included 991 patients with a diagnosis of coronary artery disease (633 men and 358 women, medium age 59.9 ± 8.8 years) who were hospitalized in Cardiology Divisions of Kursk Emergency Hospital and Vascular Surgery Division of Regional Clinical Hospital (Kursk). The following criteria were used to include patients in the case group: (1) diagnosis of coronary artery disease verified by experienced cardiologists and confirmed by clinical and instrumental methods (electrocardiography monitoring, angiography of coronary arteries); (2) CAD patients did not take lipid-lowering drugs; (3) Slavic origin (Russian, Ukrainian, or Belarusian); and (4) informed consent for participation in the study. The control group included 709 relatively healthy individuals (452 men and 257 women, mean age 60.4 ± 8.1 years) without cardiovascular and other chronic diseases. The control group was recruited for the same period of time among patients admitted at annual medical examinations, as well as among staff of medical and educational organizations. The inclusion criteria for the control group were: (1) absence of any chronic diseases, (2) Slavic origin, and (3) informed consent of a patient to participate in the study. Table 1 summarizes the demographic, clinical, and biochemical characteristics (plasma lipids) of the study participants. The case and control groups were matched according to both sex and age (*p* > 0.05). Body mass index was greater in CAD patients then in healthy subjects (*p* < 0.001). The majority of CAD patients (94.3%) suffered from hypertension, and approximately one-fifth had diabetes. The number of smokers was lower in the case group then in healthy controls (*p* = 0.002).

### 2.2. Clinical Examination of Patients

Patients with congenital heart and vascular defects, cardiomyopathies, cancer, connective tissue diseases, and chronic inflammatory diseases were not included in the case study. All study participants were interviewed for cardiovascular risk factors with a questionnaire used in our previous studies [18,19,20]. Body mass index (BMI) was calculated as the ratio of body weight in kilograms to the height in square meters (kg/m^2^). BMI of patients with coronary artery disease was 29.8 ± 5.4, that significantly exceeded (*p* < 0.0001) BMI in the control group (27.0 ± 4.5). CAD patients underwent an ultrasound examination of the brachiocephalic arteries to assess carotid intima-media thickness. CIMT was measured (in mm) using the ultrasound diagnostic system MyLab™ 40 in B-mode with an ultra-high resolution linear transducer (Esaote Europe B.V., Maastricht, The Netherlands) bilaterally in distal third of the common carotid artery at a distance of 1–1.5 cm proximal to the posterior wall bifurcation.

### 2.3. Biochemical Investigations

Venous blood samples for biochemical investigation have been taken in the morning after a 12 h fast. The following parameters of lipid metabolism in plasma were assessed: total cholesterol (TC), low-density lipoprotein cholesterol (LDL-C), high-density lipoprotein cholesterol (HDL-C), and triglycerides (TG). The level of TC and TG was determined with a direct enzymatic method on an automatic analyzer Vitalab Flexor E (Vital Scientific N.V., Spankeren, The Netherlands) using reagents Analitycon (Analyticon Biotechnologies AG, Lichtenfels, Germany). The levels of HDL-C was determined an automatic biochemical analyzer “Cobas c 311” (Roche Diagnostics GmbH, Mannheim, Germany) using a kit of reagents Roche Diagnostics. The levels of total cholesterol, LDL-C, HDL-C, and TG were expressed in mmol/L. The level of LDL-C was calculated using the Friedwald’s formula and expressed in mmol/L.

### 2.4. SNP Genotyping

Whole blood samples with 0.5M EDTA were used to isolate genomic DNA by standard phenol-chloroform extraction and ethanol precipitation. Single nucleotide polymorphisms at genes involved in the lipid metabolism were selected using public internet resources such as GWAS Catalog (www.ebi.ac.uk/gwas, accessed date 12 August 2019), PubMed (www.ncbi.nlm.nih.gov/pubmed, accessed date 4 March 2019), and HuGE Literature Finder (https://phgkb.cdc.gov/PHGKB/startPagePubLit.action, accessed date 18 April 2019), as described previously [24]. The criteria for SNP selection included: (1) SNP is associated with one or more plasma lipids such as TC, LDL-C, HDL-C, and TG at a genome-wide significance level (*p* ≤ 5 × 10^−8^), (2) association of SNP and lipid(s) was observed in European population, and (3) minor allele frequency (MAF) is ≥5%. A total 16 SNPs such as rs1883025 of *ABCA1*, rs4420638 of *APOC1*, rs3764261 of *CETP*, rs12328675 of *COBLL1*, rs3136441 of *F2*, rs4846914 of *GALNT2*, rs386000 of *LILRA3*, rs55730499 of *LPA*, rs21740 of *NPC1L1*, rs6065906 of *PLTP*, rs16942887 of *PSKH1*, rs11220463 of *ST3GAL4*, rs881844 of *STARD3*, rs1689800 of *ZNF648*, rs838880 of *SCARB1*, and rs9987289 of *PPP1R3B* (Table 2) were selected from a few dozens of lipid-associated GWAS loci. The selection of the above SNPs satisfied for their simultaneous (multiplex) detection using the iPLEX MALDI-TOF technology implemented in the MassARRAY-4 system (Agena Bioscience, San Diego, CA, USA). MassARRAY Assay Design Suite software (https://agenacx.com, accessed date 14 September 2019) was used to design the genotyping panel. Primers for genotyping were synthesized by the Evrogen company (Moscow, Russia). Primer sequences are available upon request. The quality of genotyping was assessed in 95 randomly selected DNA samples blindly to the case and control status. Repeated multiplex genotyping of these samples using the MassARRAY-4 system reached concordant results.

### 2.5. Statistical Analysis

The distribution of genotype frequencies according to the Hardy–Weinberg equilibrium (HWE) and the comparison of allele and genotype frequencies between the study groups were evaluated with Fisher’s exact test. The Kolmogorov–Smirnov test was used to assess the studied quantitative traits (i.e., plasma lipids, CIMT) for the normal distribution. Associations of SNPs with the risk of CAD were assessed by the odds ratio (OR) and 95% confidence intervals (95% CI) using multiple logistic regression with adjustments for sex, age, and BMI. Since blood lipids showed a deviation from the normal distribution (*p* < 0.05), their relationship with the polymorphisms was assessed by the Kruskal–Wallis test using software STATISTICA v13.0 (Statsoft, Tulsa, OK, USA). Plasma lipids and CIMT were expressed as medians (Me) and interquartile ranges (Q1/Q3). SNPstats statistical software [28] was used to assess the phenotypic effects of SNPs on plasma lipids and CIMT after rank-based inverse normal transformation of the traits. The impact of paired SNP combinations (diplotypes) on the normalized plasma lipids, carotid intima-media thickness, and the risk of coronary artery disease was analyzed by the likelihood-ratio test (LRT) with SNPassoc package for R [29] and adjusted for sex, age, and BMI. Replication for associations between SNPs and cardiovascular phenotypes such as coronary artery disease, TC, LDL-C, HDL-C, and TG was performed using by whole-genome genotype datasets from the Cardiovascular Knowledge Portal (https://cvd.hugeamp.org, accessed date 15 January 2022).

### 2.6. Bioinformatics Analysis

The model based multifactor dimensionality reduction (*mbmdr*) method [30,31] was used to evaluate gene-gene (G × G) and gene-environment (G × E) interactions associated with plasma lipids, CIMT and the susceptibility to coronary artery disease. *Mbmdr* is an extended variant of classic method Multifactor Dimensionality Reduction proposed by Ritchie and Hahn [32,33] for nonparametric assessment of gene–gene and gene–environment interactions underlying complex traits. Online bioinformatics tools and resources such as FuncPred (https://snpinfo.niehs.nih.gov accessed date 4 June 2019), GTEx portal (https://www.gtexportal.org, accessed date 7 June 2019), rSNPBase (http://rsnp.psych.ac.cn, accessed date 15 June 2019), and atSNP (atsnp.biostat.wisc.edu, accessed date 26 June 2019), were used for a comprehensive functional annotation of the studied polymorphisms. A web bioinformatics resource atSNP Search was used to statistically evaluate influence of human genetic variation on transcription factor binding [34]. A threshold significant *p*-value ≤ 0.01 for the SNP impact (indicates that the SNP is candidate for potential gain- or loss-of-function) was selected to assign the tested DNA motif covering a SNP area to the potential target for binding to a specific transcription factor. The molecular effects of in silico predicted transcription factors on gene expression were annotated with Gene Ontology (GO) terms (http://geneontology.org, accessed date 14 June 2019) and Uniprot database (https://www.uniprot.org, accessed date 29 June 2019). The sets of such transcription factors were enriched with GO terms to explore molecular functions, biological processes, and pathways in which these transcription factors are involved. *Enrichr* bioinformatics resource (https://maayanlab.cloud/Enrichr/, accessed date 2 July 2019) was used for the enrichment analysis.

## 3. Results

### 3.1. Association of Gene Polymorphisms with the Risk of Coronary Artery Disease, Plasma Lipids, and Carotid Intima-Media Thickness

Genotype distribution for a majority of SNPs was in Hardy–Weinberg equilibrium. However, genotype frequencies of SNPs rs3764261 of *CETP*, rs12328675 of *COBLL1*, and rs838880 of *SCARB1* showed significant deviations from those expected for HWE (*p* < 0.05). The allele frequencies of 16 lipid-associated GWAS loci in populations of Central Russia and Europe are shown in Table 3. Significant inter-population differences in MAF were found for two SNPs such as rs4420638 of *APOC1* (*p* = 0.02) and rs3136441 of *F2* (*p* < 0.0001). The frequency of allele rs3136441-C of the *F2* gene was higher in the Central Russian population than in Europeans. Table 4 shows associations of the studied polymorphisms with the risk of coronary artery disease in a population of Central Russia. As can be seen form Table 4, four of sixteen polymorphisms showed significant associations with the risk of coronary artery disease regardless of sex, age, and body mass index. In particular, SNP rs4420638 of *APOC1* was associated with an increased risk of CAD (OR = 1.49, 95% CI 1.10-2.03, *p* = 0.009, overdominant effect). Polymorphism rs55730499 of the *LPA* gene was associated with increased disease risk (OR = 1.92, 95% CI 1.30–2.83, *p* = 0.0007, recessive effect). SNPs rs3136441 of *F2* (OR = 0.49, 95% CI 0.37–0.64, *p* < 0.0001, dominant effect) and rs6065906 of *PLTP* (OR = 0.66, 95% CI 0.50–0.86, *p* = 0.002, recessive effect) showed associations with decreased disease risk.

Next, we analyzed the relationship between the studied polymorphisms and plasma lipids in CAD patients (Table 5). The normalized values of plasma lipids were used for the statistical analysis. It was found that the levels of total cholesterol are associated with SNPs such as rs12328675 of *COBLL1* (*p* = 0.02, overdominant effect), rs4846914 of *GALNT2* (*p* = 0.02, overdominant effect), rs55730499 of *LPA* (*p* = 0.03, overdominant effect), and rs838880 of *SCARB1* (*p* = 0.035, recessive effect). The levels of LDL-C were found to be associated with polymorphisms rs3764261 of *CETP* (*p* = 0.042, dominant effect), rs55730499 of *LPA* (*p* = 0.0007, recessive effect), rs1689800 of *ZNF648* (*p* = 0.026, recessive effect), and rs838880 of *SCARB1* (*p* = 0.043, overdominant effect). The levels of HDL-C showed an association with rs3764261 of the *CETP* gene (*p* = 0.005, overdominant effect). The levels of triglycerides were associated with polymorphisms rs217406 of *NPC1L1* (*p* = 0.02, recessive effect) and rs6065906 of *PLTP* (*p* = 0.03, dominant effect).

Table 6 shows associations of the studied SNPs on carotid intima-media thickness in patients with CAD. The analysis was performed on both non-transformed (Kruskal-Wallis test) and normalized (linear regression analysis) values of carotid intima-media thickness after adjustment for cofactors such as sex, age, and body mass index. As a result, three polymorphisms were established to be associated with the CIMT index in patients with coronary artery disease. In particular, SNP rs12328675 of the *COBLL1* gene was associated with a decrease in CIMT (*p* = 0.009, additive effect). The rs11220463 polymorphism of the *ST3GAL4* gene also showed association with decreased carotid intima-media thickness (*p* = 0.01, overdominant effect). However, SNP rs1689800 of *ZNF648* was associated increased CIMT in CAD patients (*p* = 0.02, dominant effect) No significant associations were found between CIMT and other gene polymorphisms.

### 3.2. Smoking as a Triggering Factor Modifying the Genetic Effects on Plasma Lipids, CIMT, and CAD Risk

The multifactorial nature of atherosclerosis and coronary artery disease means that genetic and environmental factors are jointly involved in the mechanisms underlying the disease [35,36]. In this regard, a joint analysis of genetic and environmental factors may provide deeper insights into the disease mechanisms. Hence, we analyzed associations of the studied gene polymorphisms with the risk of coronary artery disease, plasma lipids and carotid intima-media thickness in the groups stratified by smoking habit, one of the most important risk factors for cardiovascular disease. Linear regression analysis adjusted for cofactors (sex, age, and body mass index) was used to evaluate effects of the SNPs on the normalized quantitative values of plasma lipids and CIMT. The association analysis results are summarized in Table 7. Gene polymorphisms such as rs12328675 of *COBLL1* (*p* = 0.03), rs3136441 of *F2* (*p* = 0.02), and rs16942887 of *PSKH1* (*p* = 0.049) were associated with increased levels of total cholesterol in plasma exclusively in smokers. A similar trend in the associations was observed for increased levels of low-density lipoprotein cholesterol and polymorphisms rs1883025 of the *ABCA1* gene (*p* = 0.003), rs55730499 of the *LPA* gene (*p* = 0.0001), and rs1689800 of the *ZNF648* gene (*p* = 0.02) in smokers, while these associations were not established in non-smokers (*p* > 0.05).

Notably, the levels of LDL-C were increased more than two times in the carriers of genotype rs55730499-T/T of *LPA* (5.8 mmol/L) then in the carriers of genotypes C/C and C/T (2.5 mmol/L). SNP rs3764261 of the *CETP* gene was associated with increased levels of high-density lipoprotein cholesterol only in non-smokers (*p* = 0.006), while this antiatherogenic effect was not seen in smokers. A relationship between a decrease in plasma triglycerides and polymorphisms rs4846914 of *GALNT2* (*p* = 0.008) and rs386000 of *LILRA3* (*p* = 0.03) was found exclusively in non-smoking subjects. However, SNP rs4846914 of *GALNT2* was associated with increased CIMT in smokers (*p* = 0.05). On the contrary, SNPs rs217406 of *NPC1L1* (*p* = 0.02) and rs881844 of *STARD3* (*p* = 0.03) were associated with a lower CIMT, but only in non-smoker individuals. An interesting finding that SNP rs3136441 of the *F2* gene was associated with decreased risk of coronary artery disease in both smokers (*p* = 0.004) and non-smokers (*p* = 0.02). SNP rs4420638 of the *APOC1* gene was associated with increased risk of CAD in non-smokers (*p* = 0.009), while the association of this SNP in smokers showed the similar trend, but did not reach the statistical significance level (*p* = 0.10). Unexpected findings that polymorphisms rs12328675 of *COBLL1* (*p* = 0.02) and rs55730499 of *LPA* (*p* = 0.01) were associated with an increased risk of coronary artery disease in non-smokers, while no significant associations of these SNPs were found in smokers. SNP rs217406 of the *NPC1L1* gene showed significant association with an increased risk of coronary artery disease exclusively in smokers (*p* = 0.01).

### 3.3. Replication for Associations between SNPs and Cardiovacsular Phenotypes in Independent Populations

Whole-genome genotype datasets from the Cardiovascular Knowledge Portal were utilized to perform a replication study for associations between SNPs and the cardiovascular phenotypes such as coronary artery disease, TC, LDL-C, HDL-C, and TG in independent population cohorts of CAD patients and controls. Results of replication analysis were interpreted only for those genotype-phenotype associations that reached a significance *p*-value of 0.01 or lesser in our study. Table 8 shows associations of the studied polymorphisms with CAD and plasma lipids. Single nucleotide polymorphisms such as rs4420638 of *APOC1* (*p* = 1.15 × 10^−203^), rs55730499 of *LPA* (*p* = 1.45 × 10^−174^), and rs6065906 of *PLTP* (*p* = 0.01) were successfully replicated in large population cohorts as susceptibility markers for coronary artery disease. Only the rs3136441 SNP of F2 was not associated with the risk of CAD in the entire population cohort. In contrast, rs3764261 of *CETP* (*p* = 3.57 × 10^−10^), rs4846914 of *GALNT2* (*p* = 1.14 × 10^−8^) were significantly associated with the risk of CAD in independent cohorts, whereas we did not find such association in our population. Furthermore, several gene polymorphisms were successfully replicated as markers associated with plasma lipids: rs3764261 of *CETP* with HDL-C (*p* = 7.08 × 10^−36^), rs4846914 of *GALNT2* with TC (*p* = 0.049), rs55730499 of *LPA* (*p* = 4.16 × 10^−298^), and rs1689800 of *ZNF648* (*p* = 6.84 × 10^−8^) with LDL-C. In contrast to our findings, polymorphisms such as, for instance, rs1883025 of *ABCA1*, rs4420638 of *APOC1*, and rs9987289 of *PPP1R3B* showed significant associations with all studied plasma lipids in the independent populations.

### 3.4. Analysis of Pairwise SNP-SNP Interactions Contributing to the Studied Cardiovascular Phenotypes

**Figure 1:**
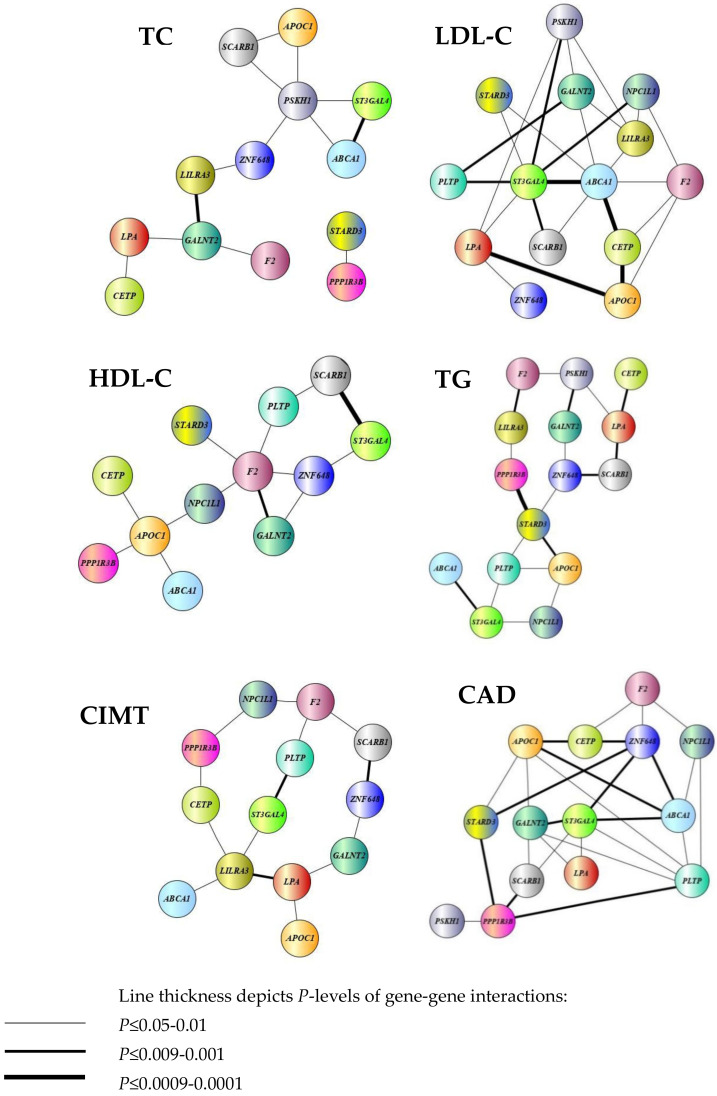
Paired interactions between genes associated with plasma lipids, CIMT, and coronary artery disease.

Polygenic mechanisms underlying atherosclerosis substantiate a need in investigating the role of gene–gene and gene–environment interactions contributing to the pathophysiology of coronary artery disease. Pursuing this interest, we analyzed joint effects of the gene polymorphisms on the cardiovascular phenotypes including plasma lipids, CIMT, as well as the risk of coronary artery disease. Initially, the LRT was used to evaluate the effects of SNP–SNP combinations on the normalized phenotypic values. The LRT analysis allowed identifying a lot of paired interactions between genes associated with plasma lipids, CIMT, and coronary artery disease. Figure 1 shows interactome networks indicating a complexity of epistatic interactions between studied gene polymorphisms significantly associated with the studied cardiovascular phenotypes. In particular, epistatic interactions between 13 gene polymorphisms influence the levels of total plasma cholesterol in CAD patients with the most strong gene–gene interactions comprising polymorphisms of *ABCA1* and *ST3GAL4*, as well as *GALNT2* and *LILRA3*. The interactome network of total plasma cholesterol shows that the *GALNT2* and *PSKH1* genes represent the nodes, each interacting with a network of other genes, and these nodes are bridged to each other through the *LILRA3* and *ZNF648* polymorphisms. In contrast, the joint effect of the *STARD3* and *PPP1R3B* SNPs on the total cholesterol levels was found regardless of other studied genes. Epistatic interactions with the most substantial effects on the plasma TC levels were found for combinations of *LILRA3* and *GALNT2* (*p* = 0.005) as well as *ABCA1* and *ST3GAL4* (*p* = 0.02).

Interactions between 14 polymorphic genes such as *ABCA1*, *APOC1*, *CETP*, *F2*, *GALNT2*, *LILRA3*, *LPA*, *NPC1L1*, *PLTP*, *PSKH1*, *ST3GAL4*, *STARD3*, *ZNF648*, and *SCARB1* showed the joint effects on cholesterol levels of atherogenic lipoproteins (i.e., LDL). Individual effects on LDL-C were established for polymorphic variants of *CETP* (*p* = 5 × 10^−5^), *LPA* (*p* = 0.002), *ZNF648* (*p* = 0.03) and *SCARB1* (*p* = 0.02). Interestingly, gene–gene interactions representing the “chain” spanned *LPA × APOC1 × CETP × ABCA1 × ST3GAL4* loci were strongest (*p*-values for the interactions varied from ≤0.0009 to 0.0001). In addition, highly significant interactions influencing C-LDL were also found between *ST3GAL4* and *ABCA1*, *SCARB1*, *PLTP*, *PSKH1*, and *NPC1L1* (*p* ≤ 0.009–0.001). Epistatic interactions between twelve genes such as *ABCA1*, *APOC1*, *CETP*, *F2*, *GALNT2*, *NPC1L1*, *PLTP*, *ST3GAL4*, *STARD3*, *ZNF648*, *SCARB1*, and *PPP1R3B* were established to impact the levels of high density lipoprotein cholesterol in plasma of CAD patients. In general, epistatic interactions involving the *F2* and *GALNT2* loci (*p* = 0.002) as well as *SCARB1* and *ST3GAL4* (*p* = 0.0003) that associated with the levels of HDL were strong. Notably, the *F2* and *APOC1* gene polymorphisms showed interactions with multiple studies SNPs that jointly affect HDL levels in plasma. Epistatic interactions between 15 polymorphic genes (*ABCA1*, *APOC1*, *CETP*, *F2*, *GALNT2*, *LILRA3*, *LPA*, *NPC1L1*, *PLTP*, *PSKH1*, *ST3GAL4*, *STARD3*, *ZNF648*, *SCARB1*, and *PPP1R3B*) were characterized by significant effects on plasma triglycerides. Individual effects of SNPs on triglycerides in CAD patients were also observed for polymorphisms of *APOC1* (*p* = 0.04) and of *NPC1L1* (*p* = 0.03). As can be seen from Figure 1, two groups of interacting genes were found to be associated with TG level in plasma: the first group included *PLTP, APOC1, NPC1L1, ST3GAL4*, and *ABCA*, whereas the second group included *F2*, *PSKH1*, *CETP*, *LPA*, *SCARB1*, *ZNF648*, *LILRA3*, *GALNT2*, and *PPP1R3B*. It is noteworthy that these groups interacted to each other through the *STARD3* polymorphism. Interactions between thirteen loci such as *ABCA1*, *APOC1*, *CETP*, *F2*, *GALNT2*, *LILRA3*, *LPA*, *NPC1L1*, *PLTP*, *ST3GAL4*, *ZNF648*, *SCARB1*, and *PPP1R3B* contributed to carotid intima-media thickness in patients with coronary artery disease. Polymorphic variants of the *APOC1* (*p* = 0.04), *ST3GAL4* (*p* = 0.01), *ZNF648* (*p* = 0.01), and *PPP1R3B* (*p* = 0.01) genes showed individual effects on CIMT. The epistatic interactions were observed between *LPA* and *LILRA3* (*p* = 0.009), *PLTP* and *ST3GAL4* (*p* = 0.001), as well as between *ZNF648* and *SCARB1* (*p* = 0.002). As can be seen from the interactome network of CIMT (Figure 1), three independent “chains” of interacting genes such as *F2 × NPC1L1 × PPP1R3B × CETP × LILRA3*, *F2 × PLTP × ST3GAL4 × LILRA3* and *F2 × SCARB1 × ZNF648 × GALNT2 × LPA × LILRA3* were associated with carotid intima-media thickness in CAD patients. Moreover, we found that the genetic susceptibility to CAD is associated with complex interactions between 14 polymorphic genes including *ABCA1*, *APOC1*, *CETP*, *F2*, *GALNT2*, *LPA*, *NPC1L1*, *PLTP*, *PSKH1*, *ST3GAL4*, *STARD3*, *ZNF648*, *SCARB1*, and *PPP1R3B*. Polymorphisms of *APOC1* (*p* = 0.01), *F2* (*p* = 8 × 10^−8^), *LPA* (*p* = 0.001), and *PLTP* (*p* = 0.0009) showed significant individual effects on the predisposition to coronary artery disease. The tight gene–gene interactions underlying CAD susceptibility comprised *GALNT2, ST3GAL4, ABCA1, ZNF648, STARD3, PPP1R3B*, and *PLTP* as well as *APOC1* and *CETP*. In particular, *PPP1R3B* interacted with *SCARB1* (*p* = 0.008) and *PLTP* (*p* = 0.005) as well as *PSKH1* (*p* = 0.02). *ST3GAL4* was found to be associated with *SCARB1* (*p* = 0.01), *LPA* (*p* = 0.02), and *PLTP* (*p* = 0.01), whereas the *F2* locus was associated with *CETP* (*p* = 0.03), *ZNF648* (*p* = 0.03), and *NPC1L1* (*p* = 0.04) gene polymorphisms. Thus, despite the fact that plasma lipids and CIMT are associated with coronary artery disease, the structure of interactions between genes that determine the studied cardiovascular phenotypes differed significantly.

### 3.5. Modeling for Gene–Gene and Gene–Environment Interactions Determining the Cardiovascular Phenotypes

**Figure 2:**
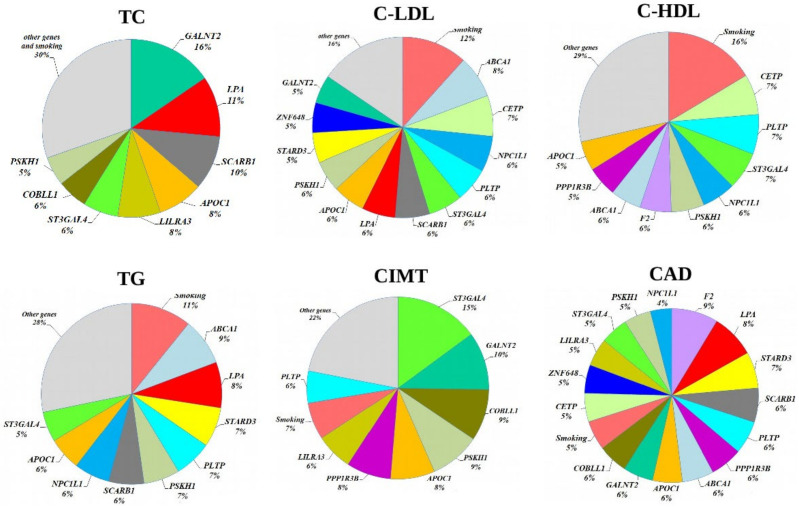
The contribution of the studied lipid-associated GWAS loci and smoking habit to the polygenic mechanisms underlying cardiovascular phenotypes.

Multifactor Dimensionality Reduction (MDR) is one of the most popular bioinformatics methods for nonparametric analysis of gene–gene and gene–environment interactions, allowing simultaneously assess to numerous variables, including gene polymorphisms, qualitative and quantitative phenotypes by reducing the dimension of the number of calculated parameters, thereby facilitating the detection of non-linear or non-additive interactions among the assessed attributes [32,33]. In the present study, we used the model-based multifactor dimensionality reduction (*mbmdr*) method allowing to detect multiple sets of significant gene–gene (G × G) and/or gene–environment (G × E) interactions in relation to a trait of interest [30,31]. Two-, three-, and four-order G × G and G × E models, including combinations of SNPs and smoking habits, were analyzed using the statistical package *mbmdr* for R, version 3.5.3 to identify associations with the levels of plasma lipids, carotid intima-media thickness, and the risk of coronary artery disease were analyzed. Six two-order, 39 three- order, and 147 four-level statistically significant MDR models (P_perm_ < 0.05) associated with the normalized blood cholesterol levels were established. The best *n*-order *mbmdr* models associated with TC, C-LDL, and CAD risk are summarized in Table 9, Table 10 and Table 11, respectively. To prioritize the attributes associated with each cardiovascular phenotype, we counted the number of *n*-models in which each attribute was involved, and the resulted value was considered as a measure of the contribution of an attribute (a variable such as SNP or smoking status) to the polygenic background, as estimated by the *mbmdr* method. Figure 2 presents diagrams illustrating the contribution (%) of each attribute to the cardiovascular phenotypes (attributes are presented in descending order of the number of models in which attributes are involved). In particular, the leading SNPs contributing to the levels of total cholesterol in plasma of CAD patients were *GALNT2* (16%), *LPA* (11%), *SCARB1* (10%), *APOC1* (8%), *LILRA3* (8%), *ST3GAL4* (6%), *COBLL1* (6%), and *PSKH1* (5%). Thus, 70% of the identified G × G *mbmdr* models associating with plasma level of TC comprised the above eight polymorphisms. It is noteworthy that the number of G × E models (SNP-smoking interactions) associated with TC was relatively small (1.7% for three-order and 4.9% for four-order *mbmdr* models). A total of 39 two-order, 281 three-order, and 1704 four-order GxG and G × E models were found to affect the normalized levels of LDL-C. As can be seen from Figure 2, the total proportion of models involving smoking status (12%) and *ABCA1* (8%), *CETP* (7%), *NPC1L1* (7%), and *PLTP* (6 %) loci was 39%. Notably, the quantity of *mbmdr* models comprising smoking status was 11% for 4-order models, 13% for 3-order models and 21% for 2-order models suggesting synergic effects tobacco smoking and these SNPs on the atherogenic fraction of lipoproteins. In total, 37 two-order, 183 three-order, and 624 four-order G × G and G × E models significantly associated with normalized HDL-C levels were identified. A significant proportion of *mbmdr* models included smoking habit: 16% for 4-order models, 15% for 3-order models, and 30% for 2-order models. In addition, 55% of the GxG and G × E models involved interactions between *CETP*, *PLTP*, *ST3GAL4*, *NPC1L1*, *PSKH1*, *F2*, *ABCA1*, *PPP1R3B*, and *APOC1* gene polymorphisms. A majority of *mbmdr* models associated with normalized levels of plasma triglycerides comprised interactions between *ABCA1* (9%), *LPA* (8%), *STARD3* (7%), *PLTP* (7%), *PSKH1* (7%), *SCARB1* (6%), *NPC1L1* (6%), *APOC1* (6%), and *ST3GAL4* (5%) loci. The *mbmdr* method allowed establishing 14 two-order, 45 three-order, and 73 four-order GxG and G × E models associated with normalized CIMT. About 70% of G × G and G × E models associated with CIMT included interactions between polymorphisms of *ST3GAL4* (15%), *GALNT2* (10%), *COBLL1* (9%), *PSKH1* (9%), *APOC1* (8%), *PPP1R3B* (8%), *LILRA3* (6%), and *PLTP* (6%) genes. Smoking status was present in 11% of the two-order, 6% of the three-order, and 7% of the four-order models. As can be seen from Table 11, the best models of G × G and G × E interactions associated with the risk of coronary artery disease included rs3136441 of *F2*, rs55730499 of *LPA*, rs881844 of *STARD3*, rs838880 of *SCARB1*, and rs12328675 of *COBLL1* gene polymorphisms. Sixteen SNPs such as *F2* (9%), *LPA* (8%), *STARD3* (7%), *SCARB1* (6%), *PLTP* (6 %), *PPP1R3B* (6%), *ABCA1* (6%), *APOC1* (6%), *GALNT2* (6%), *COBLL1* (6%), *CETP* (5%), *ZNF648* (5%), *LILRA3* (5%), *ST3GAL4* (5%), *PSKH1* (5%), and *NPC1L1* (4%) showed a comparable contribution to CAD susceptibility. Figure 2 shows the contribution of the studied lipid-associated GWAS loci and smoking to the polygenic mechanisms underlying cardiovascular phenotypes such as plasma lipids, CIMT, and coronary artery disease. A percentage of contribution for each factor was estimated as a proportion of *mbmdr* models in which a particular risk factor is involved.

**Figure 3:**
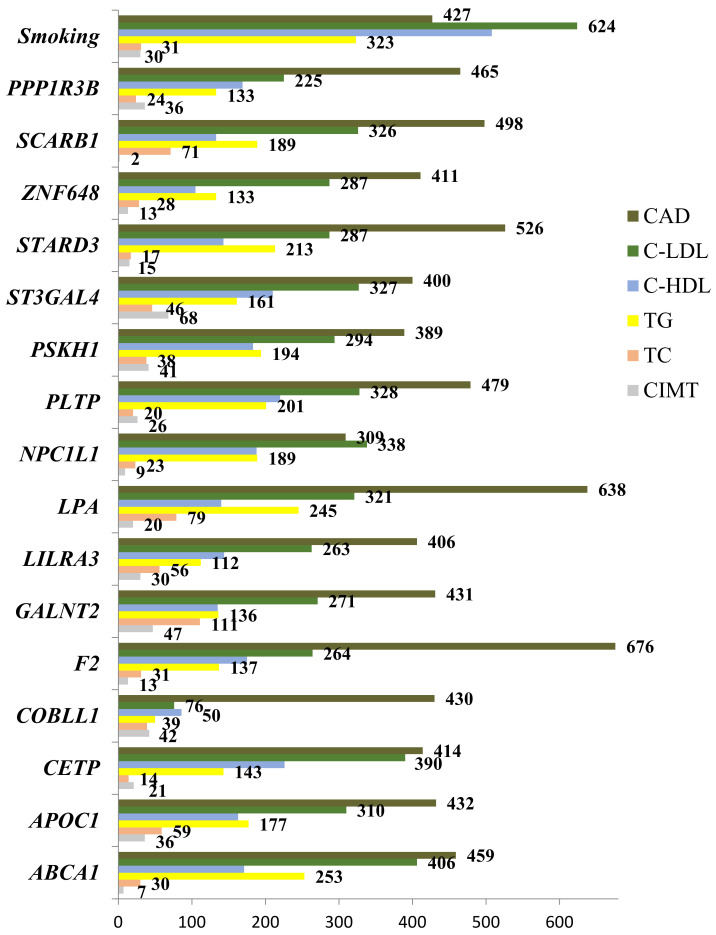
Comparative contribution of the studied gene polymorphisms and smoking habit to the cardiovascular phenotypes, as estimated by absolute number of *mbmdr* models of gene–gene and gene–smoking interactions.

A comparative contribution of each SNP and smoking status to the cardiovascular phenotypes is summarized in Figure 3. A substantial portion of associated *mbmdr* models was found for CAD. LDL-C came in the second place in terms of the number of identified *mbmdr* models. LDL-C is followed by high-density lipoprotein cholesterol and triglyceride levels. The smallest number of models was found regarding associations with carotid intima-media thickness. These findings may indicate the fact that the studied gene polymorphisms discovered by GWAS as lipid-associated loci are tightly linked to the development of coronary artery disease itself by atherogenic changes in low-density lipoprotein cholesterol rather than through other studied cardiovascular phenotypes.

### 3.6. Functional Annotation of the Studied Gene Polymorphisms

Numerous bioinformatics tools for functional SNP annotation have been developed to investigate putative molecular mechanisms by which gene polymorphisms are involved into the pathogenesis of common diseases like coronary artery disease [37,38]. In the present study, a variety of bioinformatics tools and internet resources were utilized for functional annotation of lipid-associated GWAS loci. Summary of data on the regulatory potential of the studied gene polymorphisms is shown in Table 12. As can be seen from Table 12, a majority of the studied SNPs possess a regulatory potential and represents expression quantitative trait loci (eQTLs). In particular, the regulatory potential was identified for *ABCA1*, *GALNT2*, *NPC1L1*, *STARD3*, and *PPP1R3B* polymorphisms, as in silico assessed by the FuncPred tool. Nine SNPs are associated with the levels of gene expression in coronary arteries and/or aorta. In particular, alternative alleles of *CETP* (*p* = 0.0063), *F2* (*p* = 0.0019), *LILRA3* (*p* = 4.9 × 10^−6^), *NPC1L1* (*p* = 0.0007), *PLTP* (*p* = 0.005), *PSKH1* (*p* = 0.029), and *STARD3* (*p* = 0.002) gene polymorphisms are associated with a decrease in gene expression, while the rs11220463-T allele at (*p* = 0.036) is associated with an increase in expression of the *ST3GAL4* gene. According to the rSNPbase database, many studied polymorphisms (except *APOC1*, *LILRA3*, *LPA*, and *ZNF648*) are found to be regulatory single nucleotide polymorphisms (rSNPs). Moreover, all the polymorphisms are in the linkage disequilibrium with at least one rSNPs in the genome. Notably, *PSKH1*, *STARD3*, *PPP1R3B*, *F2*, *ST3GAL4*, *GALNT2*, *PLTP*, *LILRA3*, *ABCA1*, and *NPC1L1* polymorphisms are linked to at least 10 rSNPs. The bioinformatics tool atSNP search predicted allele-specific effects of SNPs on the binding affinity of numerous transcription factors. Predicted transcription factors (TF) and their molecular effects (activator and repressor) are summarized in Supplementary Table S1. It is predicted that the alternative alleles of all SNPs create binding sites for numerous TFs (TFBS, transcription factor binding sites) that may modulate the transcriptional activity of genes. For instance, allele rs55730499-T associated with increased level of LDL-C and the risk of coronary artery disease was predicted to create binding sites for transcription factors acting as activators (NR1H, AHR::ARNT, IRF) or repressors (HAND1) of gene expression. In addition, several SNPs showed epigenetic regulatory potential. As it can be assessed with the regulatory score of RegulomeDB, the *PLTP* and *STARD3* loci have a score of *2b* (TFBS, DNA-protein interaction site, and DNAse hypersensitivity), *GALNT2*, *NPC1L1, PSKH1 ABCA1, APOC1, F2, LILRA3, SCARB1*, and *PPP1R3B* loci have scores *4* or *5* (TFBS and DNAse hypersensitivity site).

The analysis of ENCODE data showed that chromatin remodeling, in particular, chemical modification of histone H3K4me3 can regulate the access of DNA for transcriptional regulation of *PSKH1* in the aorta and *ZNF648* in adipose tissue genes through their nucleotide sequences surrounding SNPs rs16942887 and rs1689800, respectively. Histone modification H3K27ac might be associated to the transcriptional activity of the *CETP* gene in the subcutaneous adipose tissue, as well as the *PSKH1*, *ZNF648*, and *SCARB1* genes in the aorta and subcutaneous adipose tissue. Interestingly, both histone marks are found to be active enhancers regulating gene expression [39,40]. Analysis of the epigenomic data from the Roadmap Epigenomics Project allowed identifying histone modifications may affect transcriptional activity of genes in the aorta and vascular endothelium through the polymorphisms of *COBLL1*, *STARD3*, and *PPP1R3B*.

### 3.7. Enrichment Analysis of Regulatory Gene Networks in Which the Studied Gene Polymorphism Might Be Involved

**Figure 4:**
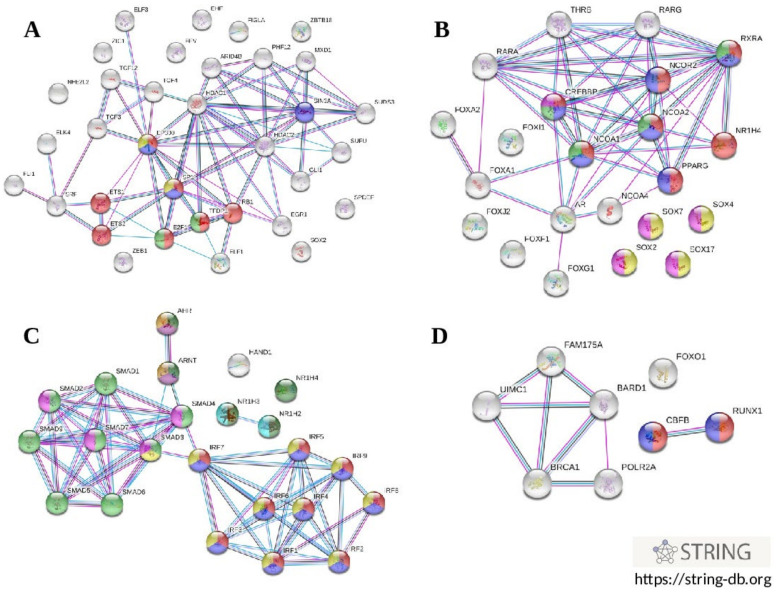
Interactomic networks involving transcription factors whose binding sites were *in silico* predicted at the alleles associated with cardiovascular phenotypes. (**A**) transcription factors associated with allele rs4420638-G of *APOC1*; (**B**) transcription factors associated with allele rs3136441-T of *F2*; (**C**), transcription factors associated with allele rs55730499-T of *LPA*; (**D**), transcription factors associated with allele rs6065906-C of *PLTP*.

Characterization of regulatory gene networks controlling the expression of genes that were identified to increase the risk of coronary artery disease has a great importance for understanding the polygenic mechanisms of the disease pathogenesis. Pursuing this interest, we first analyzed whether the sets of transcription factors that were in silico predicted to bind DNA surrounding the gene polymorphisms represent the pathways that are known to be involved in the pathogenesis of atherosclerosis. *Enrichr* bioinformatics tools (https://maayanlab.cloud/Enrichr/, accessed on 2 July 2019) accessing publicly available databases such as Gene Ontology 2018 (GO), WikiPathways 2019 Human (WP), Reactome 2016 (R-HSA), BioCarta 2016 and KEGG 2019 Human were used to identify molecular functions, biological processes, and metabolic pathways in which transcription factors might be involved. Transcription factors with predicted binding sites at the alleles associated with CAD susceptibility and/or other cardiovascular phenotypes (plasma lipids and CIMT) were a subject of interest. Molecular functions, biological processes, and metabolic pathways, which enriched with specific sets of TFs at a false discovery rate (FDR) ≤ 0.05 were considered as statistically meaningful. Transcription factors predicted to bind in the presence of the rs1883025-C allele of *ABCA1* were enriched with terms such as positive regulation of transcription, DNA-templated (GO: 0045893, FDR = 6.7 × 10^−6^), positive regulation of transcription by RNA polymerase II (GO: 0045944, FDR = 0.0002), and adipogenesis (WP236, FDR = 0.04). TFs associated with a carriage of the rs4420638 allele-G of the *APOC1* gene were enriched with terms such as positive regulation of nucleic acid-templated transcription (GO: 1903508, FDR = 2.2 × 10^−7^), positive regulation of gene expression (GO: 0010628, FDR = 9.0 × 10^−7^), positive regulation of transcription by RNA polymerase II (GO: 0045944, FDR = 0.002), and aryl hydrocarbon receptor (WP2586, FDR = 0.04). A set of TFs predicted to interact with the rs3136441-T allele of the *F2* gene were enriched with terms such as positive regulation of transcription, DNA-templated (GO: 0045893, FDR = 0.000003), positive regulation of transcription by RNA polymerase II (GO: 0045944, FDR = 0.000007), positive regulation of nucleic acid-templated transcription (GO: 1903508, FDR = 0.007), deactivation of the beta-catenin transactivating complex (R-HSA-3769402, FDR = 0.00001), and signaling by WNT (R-HSA-195721, FDR = 0.01). Transcription factors predicted to bind the rs55730499-T allele of *LPA* were enriched with numerous terms such as positive regulation of transcription by RNA polymerase II (GO: 0045944, FDR = 1.3 × 10^−20^), positive regulation of transcription, DNA-templated (GO: 0045893, FDR = 1.7 × 10^−18^), positive regulation of nucleic acid-templated transcription (GO: 1903508, FDR = 7.4 × 10^−11^), positive regulation of gene expression (GO: 0010628, FDR = 3.7 × 10^−10^), TGF-beta receptor signaling (WP560, FDR = 5.5 × 10^−13^), apoptosis (WP254, FDR = 1.4 × 10^−9^), neovascularisation (WP4331, FDR = 5.0 × 10^−9^), differentiation of white and brown adipocyte (WP2895, FDR = 0.0001), hypothesized pathways in pathogenesis of cardiovascular disease (WP3668, FDR = 0.0001), white fat cell differentiation (WP4149, FDR = 0.0002), angiogenesis (WP1539, FDR = 0.006), and adipogenesis (WP236, FDR = 0.008). Importantly, some sets of SNP-related TFs enriched with the pathways that are involved in the regulation of lipid metabolism as well as in the pathogenesis of atherosclerosis, along with the terms indicating the impact on the transcriptional activity of a gene. In particular, the rs1689800-G allele of *ZNF648* that was predicted to create binding sites for TFs enriched with a term nuclear receptors in lipid metabolism and toxicity (WP299, FDR = 0.02), whereas the rs9987289-G allele of *PPP1R3B* was associated with TFs that enriched with a term MAPK targets/nuclear events mediated by MAP kinases (R-HSA-450282, FDR = 0.04). Thus, the enrichment analysis showed that transcription factors that were in silico predicted to bind DNA motifs in the presence of CAD-associated alleles represent as transcriptional regulators capable to enhance expression of target genes as well as are involved in the regulation of lipid metabolism, inflammation and other pathways related with disease pathogenesis. Bioinformatics tools implemented in the STRING online resource (https://string-db.org, accessed on 2 July 2019) were used to visualize regulatory networks that may operate by transcription factors whose binding sites fall into the regions of the studied SNPs in the presence of alleles associated with cardiovascular phenotypes. Figure 4 shows interactomic networks of transcription factors associated with the risk alleles at SNP rs4420638 of *APOC1* (Figure 4A), rs3136441 of *F2* (Figure 4B), rs55730499 of *LPA* (Figure 4C), and rs6065906 of *PLTP* (Figure 4D). Transcription factors predicted to bind with SNP rs4420638 in the presence of allele rs4420638-G of the *APOC1* gene were enriched with Reactome ontologies such as cellular responses to stress (R-HSA-2262752, FDR = 6.4 × 10^−5^), regulation of lipid metabolism by PPARalpha (R-HSA-400206, FDR = 0.003), oxidative stress induced senescence (R-HSA-2559580, FDR = 0.03), and PPARA activates gene expression (R-HSA-1989781, FDR = 0.04). Interestingly, an interactomic network comprising transcription factors associated with allele rs3136441-T of the *F2* gene was enriched with pathways that play a role in the pathogenesis of atherosclerosis: PPARA activates gene expression (R-HSA-1989781, FDR = 1.5 × 10^−9^), transcriptional regulation of white adipocyte differentiation (R-HSA-381340, FDR = 9.8 × 10^−9^), activation of gene expression by SREBF (R-HSA-2426168, FDR = 1.1 × 10^−6^), deactivation of the beta-catenin transactivating complex (R-HSA-3769402, FDR = 1.1 × 10^−6^), and TCF dependent signaling in response to WNT (R-HSA-201681, FDR = 1.1 × 10^−5^). Transcription factors predicted to bind with SNP rs55730499 in the presence of allele T of the *LPA* gene were enriched with the following terms: interferon alpha/beta signaling (R-HSA-909733, FDR = 8.8 × 10^−15^), interferon gamma signaling (R-HSA-877300, FDR = 4.1 × 10^−14^), signaling by TGFB family members (R-HSA-9006936, FDR = 1.1 × 10^−11^), cytokine signaling in immune system (R-HSA-1280215, FDR = 2.5 × 10^−8^), TGF-beta receptor signaling activates SMADs (R-HSA-2173789, FDR = 8.6 × 10^−7^), and PPARA activates gene expression (R-HSA-1989781, FDR = 1.8 × 10^−6^). An interactomic network of transcription factors associated with allele rs6065906-C of the *PLTP* gene was enriched with terms such as RUNX1 regulates transcription of genes involved in interleukin signaling (R-HSA-8939247, FDR = 3.1 × 10^−5^), and RUNX1 regulates transcription of genes involved in WNT signaling (R-HSA-8939256, FDR = 3.6 × 10^−5^).

## 4. Discussion

### 4.1. Summary of the Study Findings and Their Comparison with Literature

The present study was designed to perform a comprehensive statistical and bioinformatics analysis to assessing the relationship between 16 single nucleotide polymorphisms that were established as markers associated with plasma lipids by genome-wide association studies (lipid-related GWAS loci) and intermediate cardiovascular phenotypes such as carotid intima-media thickness, atherogenic lipid profile as well as susceptibility to coronary artery disease. Our interest is justified by the fact that a limited number of GWAS studies have been done to assess the contribution of lipid-related loci to the risk of coronary artery disease, and few studies have performed the functional annotation of these polymorphisms, thereby complicating our understanding the mechanisms by which these loci might be involved in the pathogenesis of coronary atherosclerosis. We found that four polymorphisms such as rs4420638 of the *APOC1* gene, rs3136441 of the *F2* gene, rs55730499 of the *LPA* gene, and rs6065906 of the *PLTP* gene were significantly associated with the risk of coronary artery disease regardless of sex, age, and body mass index. Previously, several large studies found that SNP rs4420638 *APOC1* is associated with a variety of cardiometabolic and other phenotypes such as an increase in low-density lipoprotein cholesterol [11,25], increased activity of lipoprotein-associated phospholipase A2 [41], triglyceride levels [42,43], total cholesterol [44], decreased HDL cholesterol [43], increased risk of type 2 diabetes [45], Alzheimer’s disease [46,47], and age-related macular degeneration [48]. Moreover, allele rs4420638-G of *APOC1* was found to be associated with an increased risk of CAD Europeans [43], and then it was confirmed in a large meta-analysis [9]. Thus, the present study confirmed the association of SNP rs4420638 *APOC1* with an increased risk of CAD in a population of Central Russia.

It is known form the literature, allele rs3136441-C of the *F2* gene is associated with an increase in HDL-C [8]. We found that the SNP is associated with plasma levels of total cholesterol exclusively in smokers. Our study is actually the first to show the relationship between SNP rs3136441 of the *F2* gene and the development of coronary artery disease, as well as the level of total cholesterol, LDL-C and HDL-C. Allele rs55730499-T of the *LPA* gene is known to be associated with increased levels of lipoprotein (*a*) [26], coronary artery disease [9,49], and a short life expectancy [50,51]. We also confirmed the association of allele rs55730499-T of *LPA* with an increased risk of CAD in the Russian population. In addition, significant associations of this SNP with an increased level of TC and LDL-C and with a decreased level of HDL-C and TG have been identified in our study for the first time. It is known from the literature that SNP rs6065906 at *PLTP* is associated with the levels of triglycerides and HDL-C [8,44]. We also confirmed an association of this SNP with the level of triglycerides. Furthermore, our study revealed, for the first time, that the rs6065906-T of *PLTP* is associated with the increased risk of coronary artery disease. According to the summary data of GWAS catalog (https://www.ebi.ac.uk/gwas/, accessed on 2 July 2019), the rs3764261-A allele located near the *CETP* and *HERPUD1* genes is associated with an increase in the HDL cholesterol, a decrease in LDL-C and TG [8], and an increase in total cholesterol in plasma [52]. We also observed the association of allele rs3764261-A with increased levels of total blood cholesterol, but this finding occurred only in smokers. However, in contrast to previously published data, the rs3764261-A allele is associated with an increased level of LDL-C and a decreased level of HDL-C, indicting a discrepancy between ours and other studies. As can be seen from the literature, rs4846914-G allele of the *GALNT2* gene is associated with a decrease in HDL cholesterol levels and an increase in blood TG [25,44], whereas we observed an association of this SNP with an increase in total blood cholesterol levels. We also observed that the rs9987289-G allele of *PPP1R3B* is associated with increased levels of blood TC, a finding that is consistent with the results obtained by Willer with co-workers [8]. In addition, SNP rs9987289 at *PPP1R3B* is known to be associated with LDL-C and HDL-C levels [8]. We found that polymorphism rs16942887 of the *PSKH1* gene is associated with increased levels of total cholesterol in plasma exclusively in smokers, and this association is found for the first time. It is known that rs16942887 of the *PSKH1* gene is associated with HDL-C and TG levels [44,53]. We also observed a relationship between the rs838880-C allele of the *SCARB1* gene and increased levels of TC and LDL-C, the findings that are partially concordant with previously published papers. In particular, it is known that allele rs838880-C is associated with an increase in TC [54] and HDL cholesterol in plasma [8]. The present study also showed that SNP rs1883025 of the *ABCA1* gene is associated with an increase in LDL-C levels in smokers. According to the study of Hoffmann with co-authors [44], polymorphism rs1883025 SNP is also associated with LDL-C, but the association has not been investigated in subgroups of patients stratified by smoking status. SNP rs1883025 of the *ABCA1* gene is known to be associated with the levels of TC [12] and HDL-C [8]. In addition, the present study was the first to investigate relationship between the lipid-related GWAS loci and carotid intima-media thickness in patients with coronary artery disease. In particular, we found that the rs1689800-G allele of the *ZNF648* gene (locus LINC01344) is associated with an increase in LDL-C levels in smokers and increased CIMT. Interestingly, allele rs1689800-G was found to be associated with decreased levels of HDL cholesterol [8,12]. Meantime, the present study showed that polymorphisms such as rs217406 *NPC1L1* and rs881844 *STARD3* are associated with a decreased CIMT, but exclusively in non-smoker patients. The rs217406-G allele of *NPC1L1* was also linked to a decrease in blood TG levels in our study and this association was also revealed in a study of Willer with co-workers [8]. Polymorphism rs881844 at *STARD3* associated with CIMT was also associated with decreased levels of HDL-C [44,55]. We also found, for the first time, SNPs rs12328675 of *COBLL1* and rs11220463 of *ST3GAL4* are associated with a decreased CIMT. SNP rs12328675 at *COBLL1* is known to be associated with the levels of HDL-C [8] and triglycerides [44].

### 4.2. The Contribution of Gene–Gene Interactions to the Studied Cardiovascular Phenotypes

The present study showed for the first time that the interactions between lipid-related GWAS loci contribute to the risk of coronary artery disease through the characteristic changes in the lipid profile and carotid intima-media thickness. An importance of SNP-SNP interactions contributing to cardiovascular phenotypes was clearly demonstrated by the *mbmdr* method allowed identifying numerous significant models of gene–gene interactions associated with plasma lipid parameters, CIMT and coronary artery disease risk. These findings indicate the existence of epistatic interactions between lipid-associated GWAS loci, a situation when the effect of one gene may not be disclosed if the effect of another gene is not considered [56,57]. The most illustrative example for epistasis may be shown by polymorphism rs386000 of the *LILRA3* gene: this SNP was associated with none of the cardiovascular traits alone; however, this locus was present in a majority of GxG and GxE *mbmdr* models associated with the level of total blood cholesterol. Epistatic interactions between *ABCA1*, *APOC1*, *CETP*, *F2*, *GALNT2*, *LILRA3*, *LPA*, *PSKH1*, *ST3GAL4*, *STARD3*, *ZNF648*, *SCARB1*, and *PPP1R3* contributed to the level of total blood cholesterol level. At the same time, interactions between fourteen polymorphisms at genes such as *ABCA1*, *APOC1*, *CETP*, *F2*, *GALNT2*, *LILRA3*, *LPA*, *NPC1L1*, *PLTP*, *PSKH1*, *ST3GAL4*, *STARD3*, *ZNF648*, and *SCARB1* were associated with atherogenic changes in the levels of cholesterol of low density lipoproteins. Epistatic interactions between thirteen loci such as *ABCA1*, *APOC1*, *CETP*, *F2*, *GALNT2*, *LILRA3*, *LPA*, *NPC1L1*, *PLTP*, *ST3GAL4*, *ZNF648*, *SCARB1*, and *PPP1R3B* contribute to the carotid intima-media thickness in coronary artery disease patients. Although the above sets of genes were quite similar across the cardiovascular phenotypes, there were substantial differences in the structure and strength of interactions between the loci concerning particular phenotypes. The biological interpretation of such complex interactions between the genes is quite difficult and may reflect different biological phenomena, such as physical interactions between proteins, linkage disequilibrium between loci, interactions at the level of metabolism, co-expression of genes in a certain tissue, as well as shared gene regulatory networks by which transcription factors may modulate expression of genes. Nevertheless, bioinformatics methods allowed prioritizing SNP combinations whose joint effects may particularly explain the molecular mechanisms by which the lipid-associated GWAS loci are involved in the pathogenesis of coronary atherosclerosis. About 70% of G × G and G × E interaction models associated with the level of total blood cholesterol in CAD patients comprised interactions between eight loci such as *GALNT2, LPA, SCARB1, APOC1, LILRA3, ST3GAL4, COBLL1*, and *PSKH1* and certain literature data confirms the roles of these genes in the regulation of cholesterol metabolism. In particular, several studies have shown that genes such as *LPA* [44], *SCARB1* [54], *APOC1* [8], and *ST3GAL4* [44] may affect blood cholesterol levels. In addition, it is known that *GALNT2, LPA, SCARB1*, and *APOC1* are genes responsible for a hereditary form of hypercholesterolemia [58]. Interestingly, interactions between *ABCA1*, *CETP*, *NPC1L1*, *PLTP*, and cigarette smoking were associated with the levels of LDL-C. The STRING tools allowed revealing that these four proteins whose interactions were associated with the levels of LDL-C are enriched with Gene Ontologies such lipid transport (GO: 0006869), lipid metabolic process (GO: 0006629). Moreover, *ABCA1, CETP*, and *PLTP* were enriched with terms such as regulation of cholesterol efflux (GO: 0010874), plasma lipoprotein particle organization (GO: 0071827) and regulation of plasma lipoprotein particle levels (GO: 0097006). *ABCA1, CETP*, and *NPC1L1* were enriched with GO terms such as cholesterol transport (GO: 0030301) and cholesterol metabolic process (GO: 0008203). Interactions between genes such as *ABCA1*, *CETP*, *NPC1L1*, *PLTP*, *ST3GAL4 PSKH1*, *APOC1*, and smoking habit were found to influence the level of HDL-C in CAD patients. Apparently, the link of *APOC1* to these set of genes is important for the regulation of HDL-C levels since the antiatherogenic effect of this gene is attributed to the suppression of *CETP* and activation of esterified lecithin cholesterol, which plays an important role in the metabolism of esterified cholesterol between lipoproteins and cholesterol transport from peripheral tissues [59].

We observed that 70% of GxG and G × E interaction models associated with CIMT comprised polymorphic variants of the *ST3GAL4, GALNT2, COBLL1, PSKH1, APOC1, PPP1R3B, LILRA3*, and *PLTP* genes. rs12040273, another SNP of the *GALNT2* gene and the activity of plasma PLTP, have been found to be associated CIMT [60,61]. Apparently, the joint effect of *GALNT2* and *PLTP* loci on CIMT might be explained by shared pathways, in particular those regulating lipid metabolism and transport, lipid deposition in the intima of arteries, and vascular inflammation, i.e., processes that are known to influence carotid intima-media thickness [62,63]. This assumption may be supported by the finding of pleiotropic effects of some lipid-associated GWAS loci (i.e., rs11220463 *ST3GAL4*, rs4420638 *APOC1*, rs9987289 *PPP1R3B* and rs6065906 *PLTP*) on the levels of plasma lipids (i.e., TC, LDL-C, HDL-C, and TG), as well as on the levels of C-reactive protein as an inflammation marker of atherosclerosis [64]. It is important to note that the interactions between *F2* rs3136441, *LPA* rs55730499, *STARD3* rs881844, *SCARB1* rs838880, and *COBLL1* rs12328675 showed a strong contribution to the risk of coronary artery disease. It is quite obvious that each of these genes can determine independently the predisposition to coronary artery disease. For example, it is well known that polymorphic variants at the *F2* gene (coagulation factor II or thrombin) are associated with thrombosis [65,66] as well as with changes in the level of HDL-C [8,44]. *LPA* is a known independent risk factor for coronary artery disease [67], and its polymorphisms have been associated with atherogenic changes of lipid metabolism. In addition, SCARB1 may play a role as a receptor for LPA, which was found to mediate the binding and excretion of lipoprotein (*a*) [68]. It is known that polymorphisms of the *STARD3* gene are associated with the level of HDL-C [44,55], triglycerides [69], and the increased expression of *STARD3* is responsible for the antiatherogenic lipid phenotype [70]. SNPs of the *COBLL1* gene are known to be associated with various lipid-associated phenotypes, including BMI [71], waist-hip ratio [72], HDL-C levels [8], TG levels [55], and each of them is considered to be a risk factor for CAD.

### 4.3. Functional Effects of the Studied SNPs and Their Link to the Pathogenesis of Coronary Artery Disease

The preset study was the first to perform a comprehensive statistical and bioinformatics analysis aiming to better understand the mechanisms by which the established lipid-related GWAS loci are involved in the molecular mechanisms of coronary artery disease. We identified that almost all the studied SNPs, despite being located in non-coding sequences or intergenic spacers, represent functional regions of the genome that may affect gene expression in a tissue-specific manner through various molecular mechanisms. The studied lipid-associated GWAS polymorphisms may possess by own functional effects or their effects might be attributed to rSNPs that are being in the close linkage disequilibrium. Notably, a total of 393 rSNPs have been identified as to be linked to the lipid-associated GWAS loci, and these regulatory polymorphisms are located in genes that play a role in the pathogenesis of atherosclerosis. In particular, SNP rs4420638 of *APOC1* is linked to *PVRL2* (nectin cell adhesion molecule 2), *TOMM40* (translocase of the outer mitochondrial membrane 40), and *APOC4* (apolipoprotein C4). SNP rs3136441 of the *F2* gene is in a complete linkage disequilibrium with genes such as *ARHGAP1* (Rho GTPase activating protein 1), *LRP4* (protein related to LDL receptor 4), *ATG13* (protein associated with autophagy 13), *CKAP5* (protein associated with cytoskeleton 5), *ZNF408* (zinc finger protein 408), and *SNORD67* (small nucleolar RNA, C/D box 67). SNP rs6065906 of the *PLTP* gene is linked to genes such as *MMP9* (matrix metallopeptidase 9), *CTSA* (cathepsin A), *ZNF335* (zinc finger protein 335), *PCIF1* (C-terminal inhibitory factor 1 PDX1), *ZSWIM1* (SWIM-type zinc finger protein 1), *SPATA25* (protein associated with spermatogenesis 25), *NEURL2* (neuralized U3 protein ubiquitin ligase 2), *ZSWIM3* (SWIM-type 3 zinc finger protein), *FTLP* (ferritin light chain 1 pseudogene), and *ACOT8* (acyl-CoA thioesterase 8).

We identified that a half of the studied loci are *cis*- or *trans*-eQTLs, which are genome loci affecting gene expression [73]. Nine SNPs such as rs3764261 of *CETP*, rs12328675 of *COBLL1*, rs3136441 of *F2*, rs386000 of *LILRA3*, rs217406 of *NPC1L1*, rs6065906 of *PLTP*, rs16942887 of *PSKH1*, and rs11220463 of *ST3GAL4* are associated changes in gene expression in arteries and/or aorta, thereby demonstrating their putative pathogenetic involvement in atherosclerosis. The presence of *trans*-eQTLs for SNPs rs3764261 of *CETP*, rs12328675 of *COBLL1*, rs3136441 of *F2*, rs55730499 of *LPA*, rs217406 of *NPC1L1*, rs6065906 of *PLTP*, rs16942887 of *PSKH1*, and rs881844 of *STARD3* (Supplementary Table S2) may suggest that these genes are co-expressed with genes located at other regions of the genome presumably due to the shared gene regulatory networks operating by common transcription factors.

**Figure 5:**
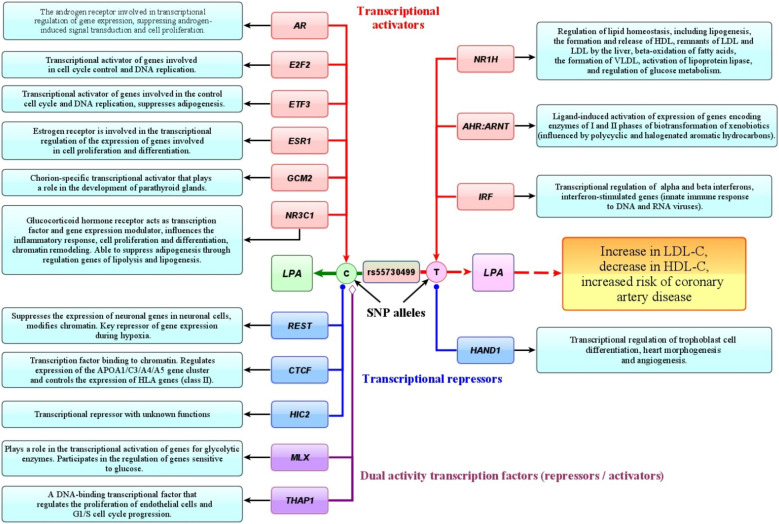
Putative effects of transcription factors whose allele-specific binding sites were in silico predicted at SNP rs55730499 of the *LPA* gene. (The color of arrows depicts the molecular effects of transcription factors (TF) on gene expression by binding to a specific SNP allele: red—transcription activators, blue—transcription repressors, purple—transcription factors with dual activity (repressors or activators). The light blue labels depict the main biological effects of TFs.)

It has been in silico predicted that many of lipid-associated GWAS loci represent the binding sites for a wide range of transcription factors capable to modulate gene expression in an allele-specific manner. Putative TFs for SNPs rs55730499 of *LPA*, rs4420638 of *APOC1*, and rs3136441 of *F2* were subjects of great interest because these loci were associated with coronary artery disease. As an example, Figure 5 summarizes the transcription factors whose binding sites are predicted at the intron region surrounding SNP rs55730499 of the *LPA* gene. An importance of this SNP for disease pathogenesis was demonstrated by two studies that observed that allele rs55730499-T of *LPA* is associated with an increased level of lipoprotein (*a*) and cholesterol of low-density lipoproteins, as well as the risk of coronary artery disease [10] and decreased life expectancy [51]. We predicted that there exist at least three transcriptional factors such as NR1H, AHR:ARNT, and IRF whose binding sites might be created by the rs55730499-T allele and enhance the expression of the *LPA* gene. Interestingly, these TFs are known as regulators of lipid metabolism, including the activation of lipogenesis, the formation and release of HDL-C by the liver, β-oxidation of fatty acids, the release of the remnants of LDL-C and LDL-C by the liver, the formation of VLDL-C, and activation of lipoprotein lipase [74,75]. Furthermore, an in silico predicted transcription factor NR1H4 is known to impact the expression of cytokine genes underlying inflammatory mechanisms of atherosclerosis [76]. The molecular complex AHR (UniProtKB—P35869):ARNT (UniProtKB—P27540) might act as another transcriptional activator affecting expression of the *LPA* gene. The AHR:ARNT complex is known to be involved in the ligand-induced cascade activation of genes encoding phase I and II biotransformation enzymes [77,78] as a result of environmental exposure to polycyclic and halogenated aromatic hydrocarbons that are chemical pollutants. Notably, it has been found by numerous studies [79,80,81] that chronic exposure to such chemicals is associated with the prevalence of atherosclerosis [82]. We suggest that the atherogenic up-regulation of *LPA* gene expression might be due to the binding of complex *AHR*:*ARNT* with a region surrounding SNP rs55730499 in the presence of allele-T associated with CAD. Transcription factor IRF3 (UniProtKB—Q14653) is involved in the transcriptional regulation of interferon α and β genes controlling the innate immune response to DNA and RNA viruses [83]. Transcriptional repressor HAND1 was predicted to bind and suppress expression of the *LPA* gene through the binding with SNP rs55730499 in the presence of the T allele. HAND1 is known to be involved in the transcriptional regulation of trophoblast cell differentiation, cardiac morphogenesis, and angiogenesis (UniProtKB—O96004).

Allele of rs55730499-C that showed protective effect against CAD susceptibility was predicted to increase an affinity to binding to six TFs such as androgen receptor AR (UniProtKB-P10275), involved in transcriptional regulation of gene expression by suppressing androgen-induced signal transduction and cell proliferation, E2F2 (UniProtKB-Q14209) activating of genes involved into the cell cycle and DNA replication, and ETF3 (UniProtKB-O00716) also activating cell cycle and DNA replication genes as well as suppressing adipogenesis. Other predicted TFs included *GCM2* (UniProtKB—O75603), a chorion-specific transcriptional activator important for the development of parathyroid glands, and NR3C1 (UniProtKB-P04150), a glucocorticoid hormone receptor acting as a transcription factor and modulator of gene expression that affects inflammatory response, chromatin remodeling, and suppresses adipogenesis through the regulation of lipolysis and lipogenesis. Transcriptional repressor REST was in silico predicted to bind with SNP rs55730499 in the presence of allele C. REST is known to be a key repressor of gene expression in hypoxia conditions by chromatin modifications that influence ischemia-induced neuronal death (UniProtKB-Q13127). We suppose that a loss of the binding site for REST due to the C > T nucleotide substitution removes the repressive “antiatherogenic” effect of this TF on expression of the *LPA* gene. Notably, the transcriptome data of the Framingham study [84] showed that the levels of *REST* expression in coronary arteries were decreased in patients with coronary artery disease, than in healthy individuals. This finding suggests the loss of binding site for REST at SNP rs55730499 may explain a loss of the “antiatherogenic” effect of this TF on the expression of the *LPA* gene. CTCF (UniProtKB-P49711) is another predicted repressor at SNP rs55730499, a chromatin-binding transcription factor regulating expression of the *APOA1*/*C3*/*A4*/*A5* gene cluster and also HLA genes of class II [85]. Finally, transcription factor THAP1 (UniProtKB-Q9NVV9) that was also in silico predicted to bind the rs55730499 SNP region is known to regulate endothelial cell proliferation and G1/S cell cycle progression, biological processes playing a role in angiogenesis [86].

It is known that *APOC1* is an important player in the metabolism of high density lipoproteins and very low-density lipoproteins (VLDL-C). The main function of *APOC1* is an inhibition of the cholesterol ester transfer protein (CETP). *APOC1* is also responsible for the activation of esterified cholesterol lecithin, which is responsible for exchanging of cholesterol between lipoproteins and its removal from peripheral tissues [87]. “Atherogenic allele” rs4420638-G of *APOC1* is recognized to be linked to the increased levels of low-density lipoprotein cholesterol [8,25], total cholesterol, and triglycerides [8,11,88], activity of lipoprotein-associated phospholipase A2 [41], as well as with life expectancy [89]. We predicted that allele rs4420638-G may create DNA binding sites for eleven transcriptional activators and two transcriptional repressors of *APOC1*. The predicted activators included EGR1, ETS1, NFE2L2, E2F1, and ETS2. EGR1 (UniProtKB-P18146) plays an important role in regulating a response of the target genes to growth factors, DNA damage, and ischemia. It also participates in the regulation of cell survival and proliferation as well as is found to mediate cellular response to ischemia and hypoxia via the regulation of expression of *IL1B* and *CXCL2*, thereby contributing to tissue inflammation and post-ischemic damage. ETS1 (UniProtKB-P14921) is a transcription factor directly controlling the expression of genes encoding cytokines and chemokines, and regulating angiogenesis through the control for migration and invasion of endothelial cells. NFE2L2 (UniProtKB-Q16236) is a transcriptional activator binding to DNA motifs (so-called ARE-elements) at the promoter of target genes involved in antioxidant defense of cells against oxidative stimuli. In fact, NFE2L2 is required for the coordinated activation of genes in the response to oxidative stress as well as for maintaining cellular redox homeostasis. E2F1 (UniProtKB-Q01094) is an activator of genes involved in the regulation of cell cycle and DNA replication, cell proliferation and TP53-dependent apoptosis. E2F1 blocks adipocyte differentiation by binding to specific promoters and inhibiting the binding of CEBPA to its target genes. ETS2 (UniProtKB-P15036) is a transcription factor that regulates expression of genes involved in human development and apoptosis. Experimental studies show that ETS2 determines the inflammatory state of endothelial cells in the severe atherosclerotic lesions of arteries [90].

The *F2* gene encodes coagulation factor II or thrombin, converting fibrinogen to fibrin and activates factors V, VII, VIII, XIII, and, in complex with thrombomodulin [91]. Moreover, F2 is a powerful vasoconstrictor and mitogen, which is the main factor contributing to vascular spasm. Thrombin is characterized by strong pro-inflammatory actions that are important for the development and progression of atherosclerosis. Acting through membrane receptors such as PAR-1, PAR-3, and PAR-4 expressed in the arterial wall, thrombin has atherogenic effects, including modulation of inflammation, migration of leukocytes into atherosclerotic plaque, increased oxidative stress, proliferation of vascular SMCs, apoptosis and angiogenesis [92,93]. The rs3136441-C allele the *F2* gene, which was found to be protective against coronary artery disease, showed association with increased levels of high-density lipoprotein cholesterol [8], and decreased expression of the *F2* gene. Two transcription repressors such as BHLHE23 and CBX5 were in silico predicted at SNP rs3136441 in the presence of allele-C. Interestingly, expression of the *BHLHE23* gene can be suppressed by environmental chemicals, including those found in the tobacco smoke [94], polycyclic aromatic hydrocarbons [95], biphenol A [96], acrylamide [97], dietary fats [98], and many of them are well known risk factors for atherosclerosis [99]. Furthermore, the atSNP tool allowed predicting that five transcription activators such as E2F2 (UniProtKB-Q14209), ETS1 (UniProtKB-P14921), ETS2 (UniProtKB-P15036) and HNF4BA (P15036) and HNF4BA (P15036), and HNF4BA (UniProt35) have an affinity for binding to a DNA motif in the presence of the rs3136441-C allele of the *F2* gene. ETS1 is a transcription factor that controlling the expression of cytokine and chemokine genes and regulating angiogenesis by the activation of genes involved in migration and invasion of endothelial cells [100]. Three transcriptional regulators such as RXRA, SOX7, and FOXA2, that have been predicted to bind with SNP rs3136441 in the presence of allele C, may have the pathogenetic role for atherosclerosis. RXRA (UniProtKB-P19793) is a retinoic acid receptor that serves as a common partner for numerous nuclear receptors. In particular, the heterodimeric complex in RXRA/PPARA is necessary to ensure the transcriptional activity of PPARA (peroxisome proliferator-activated receptor alpha) in regulating expression of genes for β-oxidation of fatty acids (*ACOX1)*, and cytochromes P450 generating free radicals leading to oxidative stress, which plays a key role in the pathogenesis of atherosclerosis. SOX7 (UniProtKB-Q9BT81) binds and activates promoter of the *CDH5* gene, playing a role in transcriptional regulation of genes expressed in vascular endothelium [101]. FOXA2 (UniProtKB-Q9Y261) is a transcription factor involved in the embryonic development and epigenetic control of gene expression by opening chromatin and accessing regulatory proteins to interact with enhancers or promoters. It is also involved in the regulation of glucose homeostasis and lipid metabolism. Interestingly, FOXA2 is also capable to bind to the promoter of fibrinogen gene (FGB), an important regulator of blood coagulation, as a result of IL6-induced activation of its transcription [102].

Enrichment analysis has identified biological processes that were enriched with several sets of transcription factors in silico predicted to bind with CAD-associated alleles at the studied GWAS loci. The enriched biological processes included adipogenesis (for TFs binding to the C-rs1883025 allele of *ABCA1*), aryl hydrocarbon receptor (for TFs binding to the G-rs4420638 allele of *APOC1*), WNT signaling (for TFs binding to the T-rs3136441 allele of *F2*), TGF-β receptor signaling, apoptosis, angiogenesis, differentiation of white and brown adipocytes, adipogenesis (for TFs binding to the T-rs55730499 of *LPA*), positive regulation of the macromolecular metabolic process and energy metabolism (for TFs binding to the C-rs881844 allele of *STARD3*), nuclear receptors in lipid metabolism and toxicity (for TFs binding to the G-rs1689800 allele of *ZNF648)* negative regulation of fat cell differentiation (for TFs that bind to the T-rs838880 allele of *SCARB1*), MAPK targets/ Nuclear events mediated by MAP kinases (for TFs binding to the G-rs9987289 allele of *PPP1R3B*). Thus, bioinformatics methods allowed establishing that lipid-related GWAS loci (1) have a regulatory potential and/or are linked to a number of regulatory polymorphisms; (2) are associated with tissue-specific gene expression; (3) are objects of epigenetic regulation of gene expression through chemical modification histones; (4) are the binding sites for transcription factors modulating gene expression. Taking together the study findings show that the phenotypic effects of the lipid-associated GWAS loci are not limited by the impact on lipid metabolism, but, most likely, are characterized by multiple or pleiotropic effects on various biological processes linking to the development of atherosclerosis. The overlapping effects of the studied genes on the cardiovascular phenotypes indicate their involvement in the polygenic mechanisms of coronary artery disease. These effects seem to be mediated by shared transcription factors representing gene regulatory networks related with the pathogenesis of atherosclerosis.

The study has some limitations. A relatively small number of participants in this study did not allow presenting more reliable estimates of the relationships between lipid-associated GWAS loci and cardiovascular phenotypes of high statistical power, as can be achieved by large-scale genome-wide association studies that we used for validating the observed genotype-phenotype correlations. Lacking some clinical data such as blood pressure, body mass index, and alcohol intake in the study patients gave us no possibility for assessing their effects as possible confounders on plasma lipid levels, carotid intima-media thickness, and the risk of coronary artery disease. Moreover, lacking data on lipid profile and CIMT in the control group did not allow replicating genotype–phenotype correlations observed in CAD patients. Because bioinformatics tools were used in this study to predict the potential effects of loci and putative disease-associated pathways, involved in the molecular mechanisms of atherosclerosis, the study findings should be considered as hypothesis-driving, and hence should be confirmed by experimental studies, including those integrating omics technologies such as genomics, transcriptomics, metabolomics, proteomics, and bioinformatics. Interpreting inter-population differences in the associations of genetic markers with cardiovascular phenotypes, we should take into account the fact that the studied polymorphisms are a priori characterized by weak or moderate phenotypic effects that are quite difficult to reproduce in independent populations, given a strong genetic heterogeneity of human populations, both in minor allele frequencies and linkage disequilibrium between the loci [103,104,105]. Finally, since the studied loci are located in noncoding regions of the human genome or intergenic spacers, their phenotypic effects in the relation to functions of neighboring genes should be interpreted with caution, since our knowledge on the mechanisms of genome function and the regulation of gene expression is still limited.

## 5. Conclusions

In the present study, we performed a comprehensive statistical and bioinformatics analysis including (a) the model-based multifactor dimensionality reduction method for stochastic modeling gene-gene and gene-environment interactions constituting the polygenic mechanisms of coronary artery disease and contributing to the intermediate cardiovascular phenotypes such as plasma lipids, lipoproteins, and carotid intima-media thickness, (b) functional SNP annotation for assessing their regulatory potential and impact on gene expression in a tissue specific manner, and (c) in silico prediction of allele-specific binding sites for transcription factors at the lipid-associated GWAS loci to identify pathways and gene regulatory networks controlling the molecular pathogenesis of CAD. The studied polymorphisms were predicted to be have significant but unequal effects on each studied phenotypes such as plasma lipids, carotid intima-media thickness, and coronary artery disease. Putative mechanisms by which lipid-associated GWAS loci contribute to CAD susceptibility involve well-recognized atherogenic changes in the plasma lipid profile, such as increased levels of total cholesterol and low-density lipoprotein cholesterol, as well as thickening of the arterial wall, as assessed through measuring CIMT. The phenotypic effects of lipid-associated GWAS polymorphisms might not be limited only by atherogenic changes in the lipid metabolism, and might involve other biological processes and metabolic pathways that play a role in atherosclerosis such as hemostasis (*F2*, coagulation factor II and *ST3GAL4*, ST3 beta-galactoside alpha-2,3-sialyltransferase 4), immune response and inflammation (*LILRA3*, leukocyte immunoglobulin like receptor A3), carbohydrate metabolic process (*PPP1R3B*, protein phosphatase 1 regulatory subunit 3B), and apoptosis (*SCARB1*, Scavenger receptor class B member 1).

The present study is the first to show that lipid-associated GWAS loci such as rs4420638 of *APOC1*, rs3136441 of *F2*, rs55730499 of *LPA* and rs6065906 of *PLTP* are susceptibility markers for coronary artery disease regardless of sex, age, and body mass index. The lipid-related GWAS loci are in tightly epistatic interactions to each other, as well as with other candidate genes contributing to atherosclerosis, thereby representing an important part of the polygenic predisposition to coronary artery disease. Finally, the methodology and approaches implemented in the present study can be used for further research focusing on deciphering the molecular mechanisms by which gene polymorphisms established by genome-wide association studies contribute to common multifactorial diseases.

## Figures and Tables

**Table 1:** Demographic, clinical, and biochemical characteristics of the study patients.

Baseline Characteristics		CAD Patients, n = 991	Healthy Controls, n = 709	*p*-Value
Age, mean ± standard deviation		59.9 ± 8.8	60.4 ± 8.1	0.23
Sex	Males, n (%)	633 (63.9)	452 (63.8)	0.96
	Females, n (%)	358 (36.1)	257 (36.2)	
Body mass index (kg/m^2^), mean ± standard deviation		29.98 ± 6.55	27.04 ± 4.48	**<0.001**
Hypertension ^1^, n (%)		935 (94.3)	0 (0.0)	-
Diabetes ^2^, n (%)		214 (21.6)	0 (0.0)	-
Smokers ^3^ (ever/never), n (%)		434 (43.9)	355 (51.5)	**0.002**
TC (mmol/L), Me (Q1; Q3)		5.76 (4.83; 6.24)	NA	-
LDL-C (mmol/L), Me (Q1; Q3)		2.20 (1.50; 3.77)	NA	-
HDL-C (mmol/L), Me (Q1; Q3)		1.30 (1.05; 1.62)	NA	-
TG (mmol/L), Me (Q1; Q3)		2.40 (1.59; 3.70)	NA	-
CIMT (mm), Me (Q1; Q3)		0.61 (0.53; 0.80)	NA	-

CAD, coronary artery disease; TC, total cholesterol; LDL-C, low-density lipoprotein cholesterol; HDL-C, high-density lipoprotein cholesterol; TG, triglycerides; CIMT, carotid intima-media thickness. Bold is statistically significant *p*-value. NA, not available. ^1^ data available from 969 CAD patients; ^2^ data available from 786 CAD patients; ^3^ data available from 988 CAD patients and 689 controls. Statistically significant *p*-values are bolded.

**Table 2:** Lipid-associated GWAS polymorphisms selected for the study.

Gene	Polymorphism (SNP ID)	SNP Location	Association of SNP with Plasma Lipids	References
*ABCA1*	C > T (rs1883025)	Intron	Decrease in TC and HDL-C	[8,25]
*APOC1*	A > G (rs4420638)	Intergenic	Increase in LDL-C	[11]
*CETP*	C > A (rs3764261)	Promoter	Increase in TC and HDL-C, decrease in LDL-C and TG	[8]
*COBLL1*	T > C (rs12328675)	3′UTR	Increase in HDL-C	[8]
*F2*	T > C (rs3136441)	Intron	Increase in HDL-C	[8]
*GALNT2*	A > G (rs4846914)	Intron	Increase in HDL-C, increase in TG	[8,25]
*LILRA3*	G > C (rs386000)	Intron	Increase in HDL-C	[8]
*LPA*	C > T (rs55730499)	Intron	Increase in Lp(a), Increaed risk of CAD	[9,26]
*NPC1L1*	C > G (rs217406)	intron	Increase in TC	[8]
*PLTP*	T > C (rs6065906)	Promoter	Decrease in HDL-Cl, increase in TG	[8]
*PSKH1*	G > A (rs16942887)	Intron	Increase in HDL-C	[8,12]
*ST3GAL4*	A > T (rs11220463)	Intron	Decrease in TC and LDL-C	[8]
*STARD3*	G > C (rs881844)	Intron	Decrease in HDL-C	[12]
*ZNF648*	A > G (rs1689800)	Intron	Decrease in HDL-C	[8,12]
*SCARB1*	T > C (rs838880)	3′UTR	Increase in HDL-C	[8]
*PPP1R3B*	G > A (rs9987289)	exon, non-coding region	Decrease in TC and HDL-C, increase in LDL-C	[8,27]

**Table 3:** Allele frequencies of the studied gene polymorphisms in European and Central Russian populations.

Gene (SNP ID)	Minor Allele	Minor Allele Frequencies (MAF) in Populations		*p*
		Central Russia (Sample Size)	European Population	
*ABCA1* (rs1883025)	T	0.222 (1697)	0.240	0.40
*APOC1* (rs4420638)	G	0.153 (1669)	0.198	**0.02**
*CETP* (rs3764261)	A	0.251 (1681)	0.292	0.07
*COBLL1* (rs12328675)	C	0.165 (1340)	0.157	0.68
*F2* (rs3136441)	C	0.205 (1675)	0.121	**<0.0001**
*GALNT2* (rs4846914)	A	0.617 (1686)	0.601	0.52
*LILRA3* (rs386000)	C	0.185 (1699)	0.191	0.76
*LPA* (rs55730499)	T	0.077 (1673)	0.076	0.94
*NPC1L1* (rs217406)	G	0.211 (1700)	0.173	0.06
*PLTP* (rs6065906)	C	0.168 (1697)	0.204	0.06
*PSKH1* (rs16942887)	A	0.122 (1678)	0.134	0.48
*ST3GAL4* (rs11220463)	T	0.166 (1699)	0.131	0.06
*STARD3* (rs881844)	G	0.663 (1693)	0.668	0.83
*ZNF648* (rs1689800)	G	0.353 (1696)	0.347	0.80
*SCARB1* (rs838880)	T	0.684 (1698)	0.687	0.90
*PPP1R3B* (rs9987289)	A	0.076 (1699)	0.075	0.94

Statistically significant *p*-values are bolded.

**Table 4:** Associations of SNPs with the risk of coronary artery disease in Central Russian population.

Gene (SNP ID)	Genotypes	Healthy Controls N (%)	CAD Patients N (%)	OR (95% CI) ^1^	*p* ^2^
*ABCA1* (rs1883025)	C/C	234 (59.2)	466 (62.4)	1.00	0.19
	C/T-T/T	161 (40.8)	281 (37.6)	0.84 (0.65–1.09)	
*APOC1* (rs4420638)	A/A-G/G	295 (78.7)	535 (71.4)	1.00	**0.009**
	A/G	80 (21.3)	214 (28.6)	**1.49 (1.10–2.03)**	
*CETP* (rs3764261)	C/C-A/A	238 (60.2)	402 (55.1)	1.00	0.17
	C/A	157 (39.8)	328 (44.9)	1.20 (0.92–1.56)	
*COBLL1* (rs12328675)	T/T-T/C	333 (93.8)	447 (90.5)	1.00	0.08
	C/C	22 (6.2)	47 (9.5)	1.61 (0.93–2.81)	
*F2* (rs3136441)	T/T	213 (54.3)	512 (69)	1.00	**<0.0001**
	T/C-C/C	179 (45.7)	230 (31)	**0.49 (0.37–0.64)**	
*GALNT2* (rs4846914)	A/A-A/G	340 (87.6)	628 (84.3)	1.00	0.17
	G/G	48 (12.4)	117 (15.7)	1.30 (0.89–1.89)	
*LILRA3* (rs386000)	G/G-G/C	382 (96.7)	728 (97.3)	1.00	0.51
	C/C	13 (3.3)	20 (2.7)	0.78 (0.37–1.63)	
*LPA* (rs55730499)	C/C	354 (89.6)	596 (81.8)	1.00	**0.0007**
	C/T-T/T	41 (10.4)	133 (18.2)	**1.92 (1.30–2.83)**	
*NPC1L1* (rs217406)	C/C-G/G	270 (68.3)	474 (63.3)	1.00	0.056
	C/G	125 (31.6)	275 (36.7)	1.30 (0.99–1.70)	
*PLTP* (rs6065906)	T/T	251 (63.5)	536 (71.8)	1.00	**0.002**
	T/C-C/C	144 (36.5)	210 (28.1)	**0.66 (0.50–0.86)**	
*PSKH1* (rs16942887)	G/G-G/A	378 (98.4)	733 (98.8)	1.00	0.47
	A/A	6 (1.6)	9 (1.2)	0.66 (0.22–1.98)	
*ST3GAL4* (rs11220463)	A/A-A/T	383 (97)	735 (98.3)	1.00	0.10
	T/T	12 (3)	13 (1.7)	0.50 (0.22–1.14)	
*STARD3* (rs881844)	G/G	155 (39.2)	333 (44.6)	1.00	0.059
	G/C-C/C	240 (60.8)	413 (55.4)	0.78 (0.60–1.01)	
*ZNF648* (rs1689800)	A/A-A/G	349 (88.3)	669 (89.6)	1.00	0.23
	G/G	46 (11.7)	78 (10.4)	0.78 (0.52–1.17)	
*SCARB1* (rs838880)	T/T	191 (48.4)	327 (43.7)	1.00	0.074
	T/C-C/C	204 (51.6)	421 (56.3)	1.26 (0.98–1.63)	
*PPP1R3B* (rs9987289)	G/G	348 (88.3)	632 (84.4)	1.00	0.062
	G/A-A/A	46 (11.7)	117 (15.6)	1.43 (0.98–2.08)	

^1^ Odds ratios and 95% confidence intervals for associations of a SNP with the risk of coronary artery disease; ^2^ *p*-values for the association of genotypes with the risk of CAD adjusted for sex, age and body mass index. Statistically significant *p*-values are bolded.

**Table 5:** Influence of polymorphisms of the studied genes on plasma lipids in CAD patients.

Gene (SNP ID)	Genotypes	Genotype Frequencies		TC			LDL-C			HDL-C			TG		
		N	%	Me	Q1/Q3 ^1^	*p* ^2^	Me	Q1/Q3 ^1^	*p* ^2^	Me	Q1/Q3 ^1^	*p* ^2^	Me	Q1/Q3 ^1^	*p* ^2^
*ABCA1* (rs1883025)	CC	386	60.9	5.72	4.82/6.27	0.67	2.13	1.50/3.54	0.19	1.32	1.04/1.63	0.09	2.46	1.67/3.72	0.26
	CT	212	33.4	5.78	4.76/6.20		2.50	1.73/3.96		1.24	1.07/1.53		1.94	1.50/3.68	
	TT	36	5.7	5.80	4.99/6.20		2.07	1.29/3.89		1.37	1.07/1.62		2.39	1.41/3.60	
*APOC1* (rs4420638)	AA	446	70.2	5.80	4.90/6.20	0.17	2.20	1.48/3.80	0.67	1.30	1.04/1.62	0.30	2.45	1.63/3.68	0.052
	AG	177	27.9	5.52	4.70/6.20		2.24	1.60/3.55		1.30	1.07/1.63		2.20	1.51/3.73	
	GG	12	1.9	5.97	5.21/6.50		2.09	1.89/3.40		1.46	1.12/1.58		3.44	1.80/4.01	
*CETP* (rs3764261)	CC	333	54.0	5.67	4.70/6.30	0.25	2.46	1.72/3.80	**0.042** **^D^**	1.24	1.02/1.58	**0.005** **^OD^**	2.37	1.59/3.70	0.17
	CA	275	44.6	5.73	4.90/6.20		2.10	1.48/3.73		1.32	1.10/1.67		2.40	1.59/3.71	
	AA	9	1.5	5.90	4.88/6.30		4.07	3.79/4.74		1.03	0.87/1.24		1.81	1.53/1.96	
*COBLL1* (rs12328675)	TT	296	75.5	5.59	4.60/6.18	**0.028** **^OD^**	3.51	2.31/4.20	0.16	1.10	0.99/1.34	0.17	1.80	1.472.69	0.06
	TC	60	15.3	5.80	4.89/6.29		3.76	2.50/4.20		1.18	1.01/1.40		1.72	1.28/2.14	
	CC	36	9.2	5.25	4.43/6.00		2.94	2.06/3.95		1.30	1.02/1.48		1.47	0.90/2.68	
*F2* (rs3136441)	TT	436	69.1	5.69	4.80/6.20	0.18	2.13	1.46/3.53	0.09	1.32	1.08/1.66	0.12	2.55	1.59/3.71	0.80
	TC	179	28.4	5.80	4.81/6.30		2.46	1.80/3.94		1.29	1.01/1.58		2.05	1.63/3.71	
	CC	16	2.5	5.89	5.20/6.20		3.27	1.90/4.08		1.05	0.92/1.14		1.93	1.50/3.50	
*GALNT2* (rs4846914)	AA	239	37.9	5.70	4.77/6.20	**0.02** **^OD^**	2.18	1.48/3.52	0.31	1.30	1.04/1.62	0.07	2.49	1.62/3.71	0.12
	AG	289	45.8	5.80	4.87/6.30		2.30	1.50/3.90		1.30	1.07/1.63		2.20	1.57/3.71	
	GG	103	16.3	5.70	4.73/6.20		2.18	1.77/3.56		1.24	1.04/1.53		2.40	1.60/3.54	
*LILRA3* (rs386000)	GG	417	65.8	5.79	4.82/6.27	0.18	2.18	1.48/3.68	0.68	1.30	1.06/1.63	0.33	2.43	1.59/3.73	0.14
	GC	198	31.2	5.70	4.87/6.20		2.24	1.70/3.85		1.26	1.04/1.60		2.38	1.62/3.68	
	CC	19	3.0	5.46	4.14/5.80		2.59	1.77/3.90		1.31	1.06/1.50		1.86	1.39/2.47	
*LPA* (rs55730499)	CC	515	82.5	5.80	4.86/6.29	**0.037** **^OD^**	2.18	1.50/3.63	**0.0007** **^R^**	1.30	1.07/1.62	0.42	2.43	1.62/3.73	0.06
	CT	108	17.3	5.43	4.59/6.17		3.30	1.80/4.00		1.20	1.00/1.44		1.88	1.34/3.04	
	TT	1	0.2	6.00	5.80/7.69		4.27	4.03/5.80		0.95	0.93/1.19		1.63	1.59/6.59	
*NPC1L1* (rs217406)	CC	375	59.1	5.80	4.81/6.27	0.47	2.30	1.54/3.88	0.63	1.30	1.05/1.62	0.09	2.40	1.61/3.70	**0.022** **^R^**
	CG	233	36.7	5.71	4.84/6.22		2.09	1.48/3.40		1.29	1.07/1.62		2.40	1.60/3.75	
	GG	27	4.3	5.40	4.89/6.00		2.20	1.75/4.02		1.28	0.91/1.59		1.92	1.51/3.12	
*PLTP* (rs6065906)	TT	449	71.0	5.70	4.81/6.24	0.50	2.20	1.51/3.72	0.17	1.30	1.07/1.62	0.052	2.24	1.57/3.53	**0.035** **^D^**
	TC	172	27.2	5.80	4.88/6.21		2.18	1.46/3.82		1.24	1.02/1.62		2.72	1.66/3.80	
	CC	11	1.7	5.90	4.98/6.20		2.82	2.06/4.06		1.10	0.96/1.40		1.92	1.57/3.75	
*PSKH1* (rs16942887)	GG	495	78.7	5.70	4.80/6.20	0.13	2.20	1.48/3.69	0.06	1.28	1.03/1.60	0.18	2.38	1.57/3.70	0.23
	GA	127	20.2	5.80	4.89/6.30		2.26	1.61/3.88		1.31	1.10/1.63		2.40	1.63/3.53	
	AA	7	1.1	6.27	5.40/6.50		3.90	2.20/4.33		1.32	0.92/1.53		2.00	1.62/3.80	
*ST3GAL4* (rs11220463)	AA	431	68.0	5.80	4.88/6.30	0.25	2.25	1.50/3.90	0.62	1.29	1.05/1.62	0.20	2.37	1.62/3.71	0.52
	AT	192	30.3	5.56	4.75/6.20		2.10	1.49/3.15		1.32	1.07/1.62		2.45	1.48/3.54	
	TT	11	1.7	5.60	4.88/6.02		2.49	1.48/4.54		0.94	0.83/1.63		3.12	2.31/4.22	
*STARD3* (rs881844)	GG	283	44.8	5.78	4.79/6.27	0.59	2.18	1.51/3.73	0.82	1.30	1.04/1.66	0.27	2.40	1.62/3.73	0.57
	GC	285	45.1	5.70	4.87/6.23		2.18	1.48/3.66		1.30	1.09/1.60		2.55	1.59/3.70	
	CC	64	10.1	5.81	4.84/6.20		2.80	1.61/4.08		1.25	1.05/1.62		1.90	1.50/3.25	
*ZNF648* (rs1689800)	AA	254	254	5.69	4.80/6.30	0.53	2.20	1.50/3.80	**0.026** **^R^**	1.31	1.03/1.58	0.18	2.44	1.62/3.74	0.62
	AG	318	318	5.80	4.86/6.20		2.10	1.45/3.40		1.30	1.07/1.66		2.41	1.59/3.70	
	GG	61	61	5.70	4.71/6.18		3.00	2.00/3.93		1.20	1.05/1.41		2.00	1.59/3.70	
*SCARB1* (rs838880)	TT	275	43.4	5.80	4.80/6.20	**0.035** **^R^**	2.31	1.69/3.93	**0.043** **^OD^**	1.27	1.04/1.62	0.65	2.20	1.59/3.50	0.30
	TC	301	47.5	5.70	4.80/6.29		2.06	1.45/3.67		1.30	1.07/1.62		2.59	1.62/3.75	
	CC	58	9.1	5.81	5.00/6.32		2.70	2.10/3.88		1.30	1.10/1.58		2.24	1.50/3.70	
*PPP1R3B* (rs9987289)	GG	538	84.7	5.70	4.81/6.20	0.10	2.18	1.49/3.74	-	1.30	1.06/1.61	-	2.45	1.60/3.71	0.51
	GA	96	15.1	5.90	5.00/6.30		2.40	1.69/3.84		1.30	1.03/1.70		2.03	1.59/3.68	
	AA	1	0.2	4.06	3.97/4.14		-	-		-	-		-	-	

^1^ medians (25/75 quartiles) of lipids in the carriers of a particular genotype; ^2^ significance level the effect of SNP on lipid parameter (transformed values), adjusted for sex, age and body mass index (linear regression analysis). R—recessive model, D—dominant model, OD—overdominance model. Statistically significant *p*-values are bolded.

**Table 6:** Impact of studied gene polymorphisms on carotid intima-media thickness in CAD patients.

Gene (SNP ID)	Genotypes	Genotype Frequencies		CIMT, mm			
		N	%	Me	Q1/Q3 ^1^	*p* ^2^	*p*_adj_^3^
*ABCA1* (rs1883025)	CC	386	60.9	0.62	0.53/0.80	0.48	0.30
	CT	212	33.4	0.60	0.52/0.79		
	TT	36	5.7	0.68	0.58/0.80		
*APOC1* (rs4420638)	AA	446	70.2	0.63	0.53/0.80	0.68	0.65
	AG	177	27.9	0.60	0.52/0.80		
	GG	12	1.9	0.61	0.55/0.63		
*CETP* (rs3764261)	CC	333	54.0	0.60	0.52/0.80	0.62	0.40
	CA	275	44.6	0.63	0.55/0.80		
	AA	9	1.5	0.57	0.57/0.57		
*COBLL1* (rs12328675)	TT	296	75.5	0.70	0.55/0.85	**0.05**	**0.009** **^R^**
	TC	60	15.3	0.65	0.50/0.78		
	CC	36	9.2	0.56	0.48/0.69		
*F2* (rs3136441)	TT	436	69.1	0.63	0.55/0.80	0.21	0.13
	TC	179	28.4	0.60	0.50/0.75		
	CC	16	2.5	0.60	0.50/0.69		
*GALNT2* (rs4846914)	AA	239	37.9	0.60	0.54/0.75	0.39	0.18
	AG	289	45.8	0.64	0.55/0.80		
	GG	103	16.3	0.61	0.50/0.85		
*LILRA3* (rs386000)	GG	417	65.8	0.60	0.53/0.80	0.49	0.13
	GC	198	31.2	0.63	0.53/0.80		
	CC	19	3.0	0.55	0.44/0.73		
*LPA* (rs55730499)	CC	515	82.5	0.62	0.53/0.80	0.31	0.31
	CT	108	17.3	0.60	0.55/0.78		
	TT	1	0.2	0.40	0.40/0.40		
*NPC1L1* (rs217406)	CC	375	59.1	0.61	0.53/0.80	0.19	0.10
	CG	233	36.7	0.62	0.54/0.80		
	GG	27	4.3	0.55	0.45/0.72		
*PLTP* (rs6065906)	TT	449	71.0	0.61	0.53/0.80	0.34	0.48
	TC	172	27.2	0.60	0.50/0.75		
	CC	11	1.7	0.79	0.63/0.85		
*PSKH1* (rs16942887)	GG	495	78.7	0.62	0.53/0.80	0.93	0.88
	GA	127	20.2	0.61	0.54/0.80		
	AA	7	1.1	0.60	0.55/0.78		
*ST3GAL4* (rs11220463)	AA	431	68.0	0.64	0.55/0.80	**0.046**	**0.01** **^OD^**
	AT	192	30.3	0.60	0.50/0.73		
	TT	11	1.7	0.55	0.55/0.80		
*STARD3* (rs881844)	GG	283	44.8	0.63	0.55/0.78	0.48	0.20
	GC	285	45.1	0.60	0.52/0.80		
	CC	64	10.1	0.57	0.50/0.78		
*ZNF648* (rs1689800)	AA	254	254	0.62	0.55/0.80	0.20	**0.02** **^D^**
	AG	318	318	0.60	0.52/0.78		
	GG	61	61	0.65	0.52/0.85		
*SCARB1* (rs838880)	TT	275	43.4	0.62	0.53/0.78	0.98	0.51
	TC	301	47.5	0.61	0.51/0.80		
	CC	58	9.1	0.60	0.53/0.78		
*PPP1R3B* (rs9987289)	GG	538	84.7	0.62	0.55/0.80	-	-
	GA	96	15.1	0.60	0.48/0.75		
	AA	1	0.2	-	-		

^1^ medians (Q1/Q3 quartiles) of lipids in the carriers of a particular genotype; ^2^ significance level for the Kruskal-Wallis test of the association of SNP with lipid parameters (non-transformed values); ^3^ significance level of the effect of SNP on lipid parameters (transformed values), adjusted for sex, age and body mass index (linear regression analysis). R—recessive model, D—dominant model, OD—overdominance model. Statistically significant *p*-values are bolded.

**Table 7:** Smoking-stratified analysis for associations of SNPs and cardiovascular phenotypes.

Gene (SNP ID)	Smoking Habit	Cardiovascular Phenotypes					
		TC	LDL-C	HDL-C	TG	CIMT	CAD
*ABCA1* (rs1883025)	Smokers	-	0.003 ^D^	-	-	-	-
	Non-smokers	-	-	-	-	-	-
*APOC1* (rs4420638)	Smokers	-	-	-	-	-	-
	Non-smokers	-	-	-	-	-	0.009 ^AD^
*CETP* (rs3764261)	Smokers	-	-	-	-	-	-
	Non-smokers	-	-	0.006 ^OD^	-	-	-
*COBLL1* (rs12328675)	Smokers	0.03 ^OD^	-	-	-	-	-
	Non-smokers	-	-	-	-	-	0.02 ^R^
*F2* (rs3136441)	Smokers	0.02 ^AD^	-	-	-	-	0.004 ^D^
	Non-smokers	-	-	-	-	-	0.02 ^R^
*GALNT2* (rs4846914)	Smokers	-	-	-	-	0.05 ^OD^	-
	Non-smokers	-	-	-	0.008 ^R^	-	-
*LILRA3* (rs386000)	Smokers	-	-	-	-	-	-
	Non-smokers	-	-	-	0.03 ^R^	-	-
*LPA* (rs55730499)	Smokers	-	0.0001 ^R^	-	-	-	-
	Non-smokers	-	-	-	-	-	0.01 ^OD^
*NPC1L1* (rs217406)	Smokers	-	-	-	-	-	0.01 ^OD^
	Non-smokers	-	-	-	-	0.02 ^R^	-
*PSKH1* (rs16942887)	Smokers	0.049 ^D^	-	-	-	-	-
	Non-smokers	-	-	-	-	-	-
*STARD3* (rs881844)	Smokers	-	-	-	-	-	-
	Non-smokers	-	-	-	-	0.03 ^R^	-
*ZNF648* (rs1689800)	Smokers	-	0.02 ^R^	-	-	-	-
	Non-smokers	-	-	-	-	-	-

*p*-values for associations were analyzed by linear (normalized TC, LDL-C, HDL-C, TG, and CIMT values) and logistic (CAD) regression analyses adjusted for sex, age and BMI. R—recessive model, D—dominant model, OD—overdominance model, AD—log-additive model. The coloured cells mean: atherogenic (pink) and anti-atherogenic (green) SNPs effects.

**Table 8:** Replication for SNP associations with coronary artery disease and studied cardiovascular phenotypes in large independent cohorts.

Gene, Effective Allele	Phenotype	*p*-Value *	Beta/Odds Ratio	Sample Size
*ABCA1* rs1883025-T	Coronary artery disease	0.0000218	▼0.9790	1,524,980
	Total cholesterol	**3.40 × 10^−91^**	▼−0.0583	431,334
	LDL cholesterol	**4.80 × 10^−43^**	▼−0.0296	682,058
	HDL cholesterol	**2.19 × 10^−139^**	▼−0.0679	385,758
	Triglycerides	**1.92 × 10^−18^**	▼−0.0190	711,468
*APOC1* rs4420638-G	Coronary artery disease	**5.80 × 10^−32^**	▲1.0814	1,477,190
	Total cholesterol	**1.15 × 10^−203^**	▲0.1357	314,177
	LDL cholesterol	**1.77 × 10^−37^**	▲0.1941	113,518
	HDL cholesterol	**1.21 × 10^−54^**	▼−0.0624	252,659
	Triglycerides	**1.06 × 10^−259^**	▲0.0605	598,528
*CETP* rs3764261-A	Coronary artery disease	**3.57 × 10^−10^**	▼0.9671	1,591,550
	Total cholesterol	**1.15 × 10^−61^**	▲0.0469	410,790
	LDL cholesterol	**1.49 × 10^−192^**	▼−0.0356	662,996
	HDL cholesterol	**7.08 × 10^−36^**	▲0.2124	14,126
	Triglycerides	**5.97 × 10^−68^**	▼−0.0362	692,195
*COBLL1* *(GRB14)* rs12328675-C	Coronary artery disease	0.004766	▼0.9835	1,481,940
	Total cholesterol	0.1169	▼−0.0097	303,083
	LDL cholesterol	0.00017	▼−0.0142	609,213
	HDL cholesterol	**3.99 × 10^−13^**	▲0.0381	315,152
	Triglycerides	**4.45 × 10^−31^**	▼−0.0406	605,928
*F2* rs3136441-C	Coronary artery disease	0.6976	▲1.0021	1,513,820
	Total cholesterol	0.0002989	▲0.0096	418,936
	LDL cholesterol	0.2641	▼−0.0031	668,268
	HDL cholesterol	**4.24 × 10^−23^**	▲0.0289	371,896
	Triglycerides	**5.12 × 10^−23^**	▼−0.0234	699,079
*GALNT2* rs4846914-G	Coronary artery disease	**1.14 × 10^−8^**	▼0.9757	1,593,010
	Total cholesterol	0.04971	▲0.0054	433,614
	LDL cholesterol	0.000917	▼−0.0071	684,307
	HDL cholesterol	**7.61 × 10^−47^**	▲0.0401	388,040
	Triglycerides	**4.74 × 10^−227^**	▼−0.0404	713,750
*LILRA3* rs386000-C	Coronary artery disease	0.5958	▲1.0029	1,448,870
	Total cholesterol	0.0000164	▲0.0143	379,606
	LDL cholesterol	0.08641	▲0.0047	624,565
	HDL cholesterol	**6.26 × 10^−23^**	▲0.0314	324,003
	Triglycerides	0.02057	▼−0.0056	663,279
*LPA* rs55730499-T	Coronary artery disease	**1.45 × 10^−174^**	▲1.3562	1,010,500
	Total cholesterol	**7.87 × 10^−11^**	▲0.0776	45,549
	LDL cholesterol	**4.16 × 10^−298^**	▲0.1235	356,869
	HDL cholesterol	0.8817	▲0.0067	45,509
	Triglycerides	**6.52 × 10^−14^**	▼−0.0357	360,181
*NPC1L1* rs217406-G	Coronary artery disease	0.0008186	▲1.0234	1,543,540
	Total cholesterol	**4.77 × 10^−13^**	▲0.0321	243,481
	LDL cholesterol	**3.23 × 10^−36^**	▲0.0364	542,160
	HDL cholesterol	0.03886	▼−0.0128	245,165
	Triglycerides	0.01255	▲0.0073	548,639
*PLTP* *(PCIF1)* rs6065906-C	Coronary artery disease	0.009999	▼0.9865	1,593,110
	Total cholesterol	0.627	▼−0.0021	380,779
	LDL cholesterol	**6.84 × 10^−8^**	▲0.0156	625,258
	HDL cholesterol	**3.62 × 10^−36^**	▼−0.0484	326,104
	Triglycerides	**1.59 × 10^−201^**	▲0.0515	663,221
*PSKH1* rs16942887-A	Coronary artery disease	0.03214	▲1.0153	1,524,990
	Total cholesterol	**2.80 × 10^−6^**	▲0.0220	385,717
	LDL cholesterol	0.7133	▲0.0015	637,521
	HDL cholesterol	**5.23 × 10^−41^**	▲0.0641	338,751
	Triglycerides	0.0000296	▼−0.0132	668,145
*ST3GAL4* rs11220463-T	Coronary artery disease	0.0000982	▲1.0234	1,591,520
	Total cholesterol	**3.52 × 10^−14^**	▲0.0220	382,747
	LDL cholesterol	**4.62 × 10^−47^**	▲0.0378	625,938
	HDL cholesterol	0.06697	▼−0.0055	326,790
	Triglycerides	0.03181	▲0.0065	665,187
*STARD3* rs881844-G	Coronary artery disease	0.0001801	▼0.9825	1,592,970
	Total cholesterol	**5.93 × 10^−6^**	▲0.0122	374,624
	LDL cholesterol	0.05305	▲0.0043	617,962
	HDL cholesterol	**4.85 × 10^−20^**	▲0.0262	318,709
	Triglycerides	0.1204	▼−0.0036	657,086
*ZNF648* rs1689800-G	Coronary artery disease	0.2033	▲1.0063	1,524,990
	Total cholesterol	0.8575	▲0.0006	433,743
	LDL cholesterol	**2.72 × 10^−9^**	▲0.0120	684,428
	HDL cholesterol	**3.72 × 10^−23^**	▼−0.0237	388,167
	Triglycerides	0.00359	▲0.0064	713,877
*SCARB1* rs838880-T	Coronary artery disease	0.0000238	▲1.0190	1,524,990
	Total cholesterol	0.0000147	▼−0.0139	292,669
	LDL cholesterol	0.6628	▲0.0009	597,781
	HDL cholesterol	**1.12 × 10^−31^**	▼−0.0316	303,498
	Triglycerides	0.03555	▲0.0053	595,585
*PPP1R3B* *(RP11-10A14.4)* rs9987289-G	Coronary artery disease	0.284	▲1.0118	1,516,240
	Total cholesterol	**3.89 × 10^−25^**	▲0.0675	373,464
	LDL cholesterol	**1.35 × 10^−47^**	▲0.0520	618,405
	HDL cholesterol	**1.72 × 10^−32^**	▲0.0640	314,374
	Triglycerides	0.01654	▼−0.0066	656,355

Genomic data obtained at the CVD Knowledge Portal (https://cvd.hugeamp.org), date of access 15 January 2022. * *p*-values reached the genome-wide significance level are bolded. ▲depicts an increased value, ▼depicts a decreased value.

**Table 9:** The best models of gene-gene and gene-environment interactions associated with total cholesterol levels in plasma of CAD patients (*mbmdr* method).

G × G/G × E Interaction Models		NH	*β*-H	WH	NL	*β*-L	WL	*P*_perm_
Two-order models								
1	*LILRA3* rs386000 × *GALNT2* rs4846914	3	0.352	17.72	0	-	-	**0.001**
2	*LPA* rs55730499 × *GALNT2* rs4846914	1	0.883	12.48	2	−0.164	5.09	**0.004**
3	*SCARB1* rs838880 × *COBLL1* rs12328675	0	-	-	2	−0.372	9.46	**0.04**
4	*SCARB1* rs838880 × *LPA* rs55730499	1	0.377	8.78	1	−2.089	4.42	**0.044**
Three-order models								
1	*SCARB1* rs838880 × *LPA* rs55730499 × *APOC1* rs4420638	3	0.436	23.04	5	−0.248	9.45	**<0.002**
2	*LPA* rs55730499 × *LILRA3* rs386000 × *GALNT2* rs4846914	4	0.549	22.04	3	−0.287	9.45	**<0.002**
3	*LILRA3* rs386000 × *GALNT2* rs4846914 × SMOKING	4	0.426	21.87	1	−0.443	3.13	**<0.002**
4	*LPA* rs55730499 × *GALNT2* rs4846914× *ABCA1* rs1883025	4	0.947	20.61	3	−0.199	6.14	**<0.002**
Four-order models								
1	*LILRA3* rs386000 × *GALNT2* rs4846914 × *COBLL1* rs12328675 × SMOKING	6	0.688	32.02	2	−0.859	12.06	**<0.002**
2	*STARD3* rs881844 × *ST3GAL4* rs11220463× *LPA* rs55730499 × *APOC1* rs4420638	5	0.544	25.21	2	−0.206	4.43	**<0.002**
3	*SCARB1* rs838880 × *LPA* rs55730499 × *APOC1* rs4420638 × SMOKING	4	0.562	23.56	4	−0.870	12.15	**<0.002**
4	*SCARB1* rs838880 × *LILRA3* rs386000 × *GALNT2* rs4846914 × *COBLL1* rs12328675	6	0.856	35.96	3	−0.759	13.91	**0.002**

NH—number of interacting genotypes / high-risk environmental factors; *β*-H—regression coefficient for high-risk interactions identified in step 2 of the analysis. WH—Wald statistics for high-risk interactions; NL is the number of interacting genotypes / low risk environmental factors; *β*-L—regression coefficient for low-risk interactions identified in step 2 of the analysis; WL—Wald statistics for low-risk interactions. *P*_perm_—permutation significance levels for the models (all models are adjusted for sex, age, and BMI). *Mbmdr*, model based multifactor dimensionality reduction method [30,31]. Statistically significant *p*-values are bolded.

**Table 10:** The best models of gene-gene and gene-environment interactions associated with LDL cholesterol in plasma of CAD patients (*mbmdr* method).

G × G/G × E Interaction Models		NH	*β*-H	WH	NL	*β*-L	WL	*P*_perm_
Two-order models								
1	*PSKH1* rs16942887 × SMOKING	2	0.596	47.25	2	−0.561	40.94	**<0.001**
2	*ABCA1* rs1883025 × SMOKING	3	0.569	43.18	3	−0.569	43.18	**<0.001**
3	*ST3GAL4* rs11220463 × SMOKING	2	0.563	42.46	3	−0.566	42.68	**<0.001**
4	*SCARB1* rs838880 × SMOKING	2	0.547	40.48	3	−0.566	42.68	**<0.001**
Three-order models								
1	*PSKH1* rs16942887 × *F2* rs3136441 × SMOKING	3	0.626	53.01	3	−0.498	28.45	**<0.002**
2	*ABCA1* rs1883025 × *PLTP* rs6065906 × SMOKING	3	0.609	51.58	3	−0.619	39.64	**<0.002**
3	*PSKH1* rs16942887 × *LILRA3* rs386000 × SMOKING	4	0.615	51.45	3	−0.521	33.49	**<0.002**
4	*SCARB1* rs838880 × *ABCA1* rs1883025 × SMOKING	4	0.532	37.84	7	−0.663	50.48	**<0.002**
Four-order models								
1	*SCARB1* rs838880 × *PSKH1* rs16942887 × *NPC1L1* rs217406 × SMOKING	7	0.662	57.46	7	−0.685	47.82	**<0.002**
2	*STARD3* rs881844 × *NPC1L1* rs217406 × *LILRA3* rs386000 × SMOKING	6	0.681	56.28	4	−0.547	19.32	**<0.002**
3	*SCARB1* rs838880 × *ST3GAL4* rs11220463 × *GALNT2* rs4846914 × SMOKING	3	0.473	17.22	10	−0.696	55.97	**<0.002**
4	*PSKH1* rs16942887 × *PLTP* rs6065906 × *F2* rs3136441 × SMOKING	5	0.648	55.90	7	−0.579	36.11	**<0.002**

NH—number of interacting genotypes/high-risk environmental factors; *β*-H—regression coefficient for high-risk interactions identified in step 2 of the analysis. WH—Wald statistics for high-risk interactions; NL is the number of interacting genotypes/low risk environmental factors; *β*-L—regression coefficient for low-risk interactions identified in step 2 of the analysis; WL—Wald statistics for low-risk interactions. *P*_perm_—permutation significance levels for the models (all models are adjusted for sex, age, and BMI). *Mbmdr*, model based multifactor dimensionality reduction method [30,31]. Statistically significant *p*-values are bolded.

**Table 11:** The best models of gene-gene and gene-environment interactions associated with CAD susceptibility (*mbmdr* method).

G × G/G × E Interaction Models		NH	*β*-H	WH	NL	*β*-L	WL	*P*_perm_
Two-order models								
1	*LPA* rs55730499 × *COBLL1* rs12328675	2	0.220	41.65	2	−0.209	43.84	**<0.001**
2	*STARD3* rs881844 × *F2* rs3136441	1	0.119	20.13	4	−0.161	40.10	**<0.001**
3	*LILRA3* rs386000 × *F2* rs3136441	2	0.142	33.29	3	−0.128	25.97	**<0.001**
4	*LPA* rs55730499 × *F2* rs3136441	3	0.133	28.12	2	−0.150	33.18	**<0.001**
Three-order models								
1	*PLTP* rs6065906 × *LPA* rs55730499 × *COBLL1* rs12328675	3	0.280	52.83	3	−0.099	13.26	**<0.002**
2	*LPA* rs55730499 × *COBLL1* rs12328675 × SMOKING	3	0.265	51.27	2	−0.104	13.95	**<0.002**
3	*LPA* rs55730499 × *LILRA3* rs386000 × *COBLL1* rs12328675	4	0.253	50.63	3	−0.132	23.58	**<0.002**
4	*SCARB1* rs838880 × *LPA* rs55730499 × *COBLL1* rs12328675	4	0.277	48.58	2	−0.127	19.67	**<0.002**
Four-order models								
1	*SCARB1* rs838880 × *STARD3* rs881844 × *LPA* rs55730499 × *COBLL1* rs12328675	9	0.345	68.48	4	−0.157	29.20	**<0.002**
2	*SCARB1* rs838880 × *LPA* rs55730499 × *COBLL1* rs12328675 × SMOKING	6	0.331	67.94	2	−0.147	16.68	**<0.002**
3	*STARD3* rs881844 × *PLTP* rs6065906 × *NPC1L1* rs217406 *NPC1L1* × *F2* rs3136441	3	0.139	16.98	12	−0.221	64.49	**<0.002**
4	*LPA* rs55730499 × *F2* rs3136441 *COBLL1* rs12328675 × SMOKING	6	0.312	63.97	5	−0.189	38.10	**<0.002**

NH—number of interacting genotypes/high-risk environmental factors; *β*-H—regression coefficient for high-risk interactions identified in step 2 of the analysis. WH—Wald statistics for high-risk interactions; NL is the number of interacting genotypes/low risk environmental factors; *β*-L—regression coefficient for low-risk interactions identified in step 2 of the analysis; WL—Wald statistics for low-risk interactions. *P*_perm_—permutation significance levels for the models (all models are adjusted for sex, age, and BMI). *Mbmdr*, model based multifactor dimensionality reduction method [30,31]. Statistically significant *p*-values are bolded.

**Table 12:** Summary of data on the regulatory potential of the studied gene polymorphisms.

SNP ID	Gene	FuncPred ^1^		Number eQTL (GTEx ^2^)		Binding Sites for TF ^3^		Regulatory Potential (rSNPbase ^4^)			
		Regulatory Potential	Conservatism	*cis*	*trans*	Loss	Gain	rSNP	rSNP in LD	Post-Transcriptional Regulation	Circular RNA Binding Regions (circRNA)
rs1883025	*ABCA1*	0.149	0.001	−/−	-	24	9	+	18	+	5
rs4420638	*APOC1*	-	0	−/−	-	2	25	-	7	-	4
rs3764261	*CETP*	0	0.001	−/+	1	11	13	+	1	-	-
rs12328675	*COBLL1*	0	0	−/+	1	8	10	+	3	-	1
rs3136441	*F2*	-	0.017	−/+	33	13	8	+	49	+	1
rs4846914	*GALNT2*	0.199	0	−/−	-	5	7	+	21	+	3
rs386000	*LILRA3*	0	0.001	26/+	-	-	-	-	19	-	10
rs55730499	*LPA*	-	-	−/−	1	11	5	-	2	-	3
rs217406	*NPC1L1*	0.339	0	1/+	17	9	7	+	18	+	4
rs6065906	*PLTP*	-	0	13/+	7	41	3	+	20	-	-
rs16942887	*PSKH1*	-	0.006	−/+	45	12	4	+	81	+	-
rs11220463	*ST3GAL4*	0	0.001	8/+	-	19	9	+	24	+	-
rs881844	*STARD3*	0.273	0.002	1/+	38	26	11	+	73	+	1
rs1689800	*ZNF648*	0	0	−/−	-	8	21	-	5	-	-
rs838880	*SCARB1*	-	0	−/−	-	3	22	+	1	-	-
rs9987289	*PPP1R3B*	0.045	0.001	−/−	-	9	26	+	51	-	-

^1^ FuncPred, online SNP function prediction tool of National Institute of Environmental Health Sciences (https://snpinfo.niehs.nih.gov/snpinfo/snpfunc.html, accessed on 2 July 2019); ^2^ GTEx portal, portal of tissue-specific gene expression and regulation, Broad Institute (https://gtexportal.org, accessed on 2 July 2019). The numerator (column *cis*) represents the total number of eQTL, the denominator is the presence (+) or absence (-) of eQTL in the aorta and coronary arteries; ^3^ atSNP, a web resource for statistically evaluating influence of human genetic variation on transcription factor binding (http://atsnp.biostat.wisc.edu accessed on 2 July 2019); ^4^ rSNPbase, a database for curated regulatory SNPs (http://rsnp.psych.ac.cn accessed on 2 July 2019).

## Data Availability

Data supporting reported results are available upon request.

## Supplementary Materials

The following are available online at https://www.mdpi.com/article/10.3390/biomedicines10020259/s1. Table S1: Binding sites of transcription factors predicted at the studied gene polymorphisms; Table S2: Trans-QTLs for eight lipid-associated GWAS polymorphisms.

## References

[1] (2017). Cardiovascular Diseases (CVDs) WHO Fact Sheet. https://www.who.int/ru/news-room/fact-sheets/detail/cardiovascular-diseases-(cvds).

[2] Roger V.L., Go A.S., Lloyd-Jones D.M., Benjamin E.J., Berry J.D., Borden W.B., Bravata D.M., Dai S., Ford E.S., Fox C.S. (2012). Heart Disease and Stroke Statistics—2012 Update: A Report from the American Heart Association. Circulation.

[3] Gofman J.W., Lindgren F., Elliott H., Mantz W., Hewitt J., Strisower B., Herring V., Lyon T.P. (2019). The Role of Lipids and Lipoproteins in Atherosclerosis. https://www.ncbi.nlm.nih.gov/books/NBK343489/.

[4] Jin J.-L., Zhang H.-W., Cao Y.-X., Liu H.-H., Hua Q., Li Y.-F., Zhang Y., Wu N.-Q., Zhu C.-G., Xu R.-X. (2020). Association of small dense low-density lipoprotein with cardiovascular outcome in patients with coronary artery disease and diabetes: A prospective, observational cohort study. Cardiovasc. Diabetol..

[5] Zhang H.-W., Jin J.-L., Cao Y.-X., Liu H.-H., Zhang Y., Guo Y.-L., Wu N.-Q., Zhu C.-G., Gao Y., Xu R.-X. (2020). Association of small dense LDL-cholesterol with disease severity, hypertension status and clinical outcome in patients with coronary artery disease. J. Hypertens..

[6] Nezu T., Hosomi N., Aoki S., Matsumoto M. (2016). Carotid Intima-Media Thickness for Atherosclerosis. J. Atheroscler. Thromb..

[7] Khera A.V., Kathiresan A.V.K.S. (2017). Genetics of coronary artery disease: Discovery, biology and clinical translation. Nat. Rev. Genet..

[8] Willer C.J., Schmidt E.M., Sengupta S., Peloso G.M., Gustafsson S., Kanoni S., Ganna A., Chen J., Buchkovich M.L., Mora S. (2013). Discovery and refinement of loci associated with lipid levels. Nat. Genet..

[9] Nikpay M., Goel A., Won H.H., Hall L.M., Willenborg C., Kanoni S., Saleheen D., Kyriakou T., Nelson C.P., Hopewell J.C. (2015). A comprehensive 1000 Genomes–based genome-wide association meta-analysis of coronary artery disease. Nat. Genet..

[10] Welter D., MacArthur J., Morales J., Burdett A., Hall P., Junkins H., Klemm A., Flicek P., Manolio T., Hindorff L. (2013). The NHGRI GWAS Catalog, a curated resource of SNP-trait associations. Nucleic Acids Res..

[11] Willer C.J., Sanna S., Jackson A.U., Scuteri A., Bonnycastle L.L., Clarke R., Heath S.C., Timpson N.J., Najjar S.S., Stringham H.M. (2008). Newly identified loci that influence lipid concentrations and risk of coronary artery disease. Nat. Genet..

[12] Teslovich T.M., Musunuru K., Smith A.V., Edmondson A.C., Stylianou I.M., Koseki M., Pirruccello J.P., Ripatti S., Chasman D.I., Willer C.J. (2010). Biological, clinical and population relevance of 95 loci for blood lipids. Nature.

[13] Chasman D.I., Pare G., Zee R.Y., Parker A.N., Cook N.R., Buring J.E., Kwiatkowski D.J., Rose L.M., Smith J.D., Williams P.T. (2008). Genetic Loci Associated with Plasma Concentration of Low-Density Lipoprotein Cholesterol, High-Density Lipoprotein Cholesterol, Triglycerides, Apolipoprotein A1, and Apolipoprotein B Among 6382 White Women in Genome-Wide Analysis with Replication. Circ. Cardiovasc. Genet..

[14] Takeuchi F., Isono M., Katsuya T., Yokota M., Yamamoto K., Nabika T., Shimokawa K., Nakashima E., Sugiyama T., Rakugi H. (2012). Association of Genetic Variants Influencing Lipid Levels with Coronary Artery Disease in Japanese Individuals. PLoS ONE.

[15] Mirzaev K., Abdullaev S., Akmalova K., Sozaeva J., Grishina E., Shuev G., Bolieva L., Sozaeva M., Zhuchkova S., Gimaldinova N. (2020). Interethnic differences in the prevalence of main cardiovascular pharmacogenetic biomarkers. Pharmacogenomics.

[16] Heinig M. (2018). Using Gene Expression to Annotate Cardiovascular GWAS Loci. Front. Cardiovasc. Med..

[17] Polonikov A., Solodilova M., Ivanov V.P., Shestakov A.M., Ushachev D.V., Vialykh E.K., Vasil’Eva O.V., Poliakova N.V., Antsupov V.V., Kabanina V. (2011). A protective effect of GLY272SER polymorphism of GNB3 gene in development of essential hypertension and its relations with environmental hypertension risk factors. Ter. Arkh..

[18] Polonikov A.V., Ushachev D.V., Ivanov V.P., Churnosov M.I., Freidin M.B., Ataman A.V., Harbuzova V.Y., Bykanova M.A., Bushueva O.Y., Solodilova M.A. (2015). Altered erythrocyte membrane protein composition mirrors pleiotropic effects of hypertension susceptibility genes and disease pathogenesis. J. Hypertens..

[19] Polonikov A., Kharchenko A., Bykanova M., Sirotina S., Ponomarenko I., Bocharova A., Vagaytseva K., Stepanov V., Bushueva O., Churnosov M. (2017). Polymorphisms of CYP2C8, CYP2C9 and CYP2C19 and risk of coronary heart disease in Russian population. Gene.

[20] Sirotina S., Ponomarenko I., Kharchenko A., Bykanova M., Bocharova A., Vagaytseva K., Stepanov V., Churnosov M., Solodilova M., Polonikov A. (2018). A Novel Polymorphism in the Promoter of theCYP4A11Gene Is Associated with Susceptibility to Coronary Artery Disease. Dis. Markers.

[21] Azarova I.E., Klyosova E.Y., Lazarenko V.A., Konoplya A.I., Polonikov A.V. (2020). rs11927381 Polymorphism and Type 2 Diabetes Mellitus: Contribution of Smoking to the Realization of Susceptibility to the Disease. Bull. Exp. Biol. Med..

[22] Medvedeva M.V. (2021). Associations of rs2305948 and rs1870377 polymorphic variants of the vascular endothelial growth factor receptor type 2 (KDR) gene with the risk of coronary heart disease. Res. Results Biomed..

[23] Kononov S., Mal G., Azarova I., Klyosova E., Bykanova M., Churnosov M., Polonikov A. (2022). Pharmacogenetic loci for rosuvastatin are associated with intima-media thickness change and coronary artery disease risk. Pharmacogenomics.

[24] Polonikov A.V., Klyosova E.Y., Azarova I.E., Kursk State Medical University (2021). Bioinformatic tools and internet resources for functional annotation of polymorphic loci detected by genome wide association studies of multifactorial diseases (review). Res. Results Biomed..

[25] Kathiresan S., Melander O., Guiducci C., Surti A., Burtt N.P., Rieder M.J., Cooper G.M., Roos C., Voight B.F., Havulinna A.S. (2008). Six new loci associated with blood low-density lipoprotein cholesterol, high-density lipoprotein cholesterol or triglycerides in humans. Nat. Genet..

[26] Mack S., Coassin S., Rueedi R., Yousri N.A., Seppälä I., Gieger C., Schönherr S., Forer L., Erhart G., Marques-Vidal P. (2017). A genome-wide association meta-analysis on lipoprotein (a) concentrations adjusted for apolipoprotein (a) isoforms. J. Lipid Res..

[27] Zhang Y., Gan W., Tian C., Li H., Lin X., Chen Y. (2013). Association ofPPP1R3Bpolymorphisms with blood lipid and C-reactive protein levels in a Chinese population. J. Diabetes.

[28] Solé X., Guinó E., Valls J., Iniesta R., Moreno V. (2006). SNPStats: A web tool for the analysis of association studies. Bioinformatics.

[29] González J.R., Armengol L., Solé X., Guinó E., Mercader J.M., Estivill X., Moreno V. (2007). SNPassoc: An R package to perform whole genome association studies. Bioinformatics.

[30] Calle M.L., Urrea V., Vellalta G., Malats N., Steen K.V. (2008). Improving strategies for detecting genetic patterns of disease susceptibility in association studies. Stat. Med..

[31] Calle M.L., Urrea V., Malats N., Van Steen K. (2010). mbmdr: An R package for exploring gene–gene interactions associated with binary or quantitative traits. Bioinformatics.

[32] Ritchie M.D., Hahn L.W., Roodi N., Bailey L.R., Dupont W.D., Parl F.F., Moore J.H. (2001). Multifactor-Dimensionality Reduction Reveals High-Order Interactions among Estrogen-Metabolism Genes in Sporadic Breast Cancer. Am. J. Hum. Genet..

[33] Hahn L.W., Ritchie M.D., Moore J.H. (2003). Multifactor dimensionality reduction software for detecting gene-gene and gene-environment interactions. Bioinformatics.

[34] Shin S., Hudson R., Harrison C., Craven M., Keleş S. (2018). atSNP Search: A web resource for statistically evaluating influence of human genetic variation on transcription factor binding. Bioinformatics.

[35] Sing C.F., Stengârd J.H., Kardia S.L. (2003). Genes, Environment, and Cardiovascular Disease. Arter. Thromb. Vasc. Biol..

[36] Lippi G., Cervellin G. (2016). The interplay between genetics, epigenetics and environment in modulating the risk of coronary heart disease. Ann. Transl. Med..

[37] Teufel A., Krupp M., Weinmann A., Galle P.R. (2006). Current bioinformatics tools in genomic biomedical research (Review). Int. J. Mol. Med..

[38] Manzoni C., Kia D.A., Vandrovcova J., Hardy J., Wood N., Lewis P.A., Ferrari R. (2018). Genome, transcriptome and proteome: The rise of omics data and their integration in biomedical sciences. Briefings Bioinform..

[39] Wysocka J., Swigut T., Xiao H., Milne T., Kwon S.Y., Landry J., Kauer M., Tackett A.J., Chait B.T., Badenhorst P. (2006). A PHD finger of NURF couples histone H3 lysine 4 trimethylation with chromatin remodelling. Nature.

[40] Creyghton M.P., Cheng A.W., Welstead G.G., Kooistra T., Carey B.W., Steine E.J., Hanna J., Lodato M.A., Frampton G.M., Sharp P.A. (2010). Histone H3K27ac separates active from poised enhancers and predicts developmental state. Proc. Natl. Acad. Sci. USA.

[41] Suchindran S., Rivedal D., Guyton J.R., Milledge T., Gao X., Benjamin A., Rowell J., Ginsburg G.S., McCarthy J.J. (2010). Genome-Wide Association Study of Lp-PLA2 Activity and Mass in the Framingham Heart Study. PLoS Genet..

[42] Saxena R., Voight B.F., Lyssenko V., Burtt N.P., de Bakker P.I.W., Chen H., Roix J.J., Kathiresan S., Hirschhorn J.N., Daly M.J. (2007). Genome-Wide Association Analysis Identifies Loci for Type 2 Diabetes and Triglyceride Levels. Science.

[43] Waterworth D.M., Ricketts S.L., Song K., Chen L., Zhao J.H., Ripatti S., Aulchenko Y., Zhang W., Yuan X., Lim N. (2010). Genetic Variants Influencing Circulating Lipid Levels and Risk of Coronary Artery Disease. Arter. Thromb. Vasc. Biol..

[44] Hoffmann T.J., Theusch E., Haldar T., Ranatunga D.K., Jorgenson E., Medina M.W., Kvale M.N., Kwok P.-Y., Schaefer C., Krauss R.M. (2018). A large electronic-health-record-based genome-wide study of serum lipids. Nat. Genet..

[45] Zhao W., Rasheed A., Tikkanen E., Lee J.-J., Butterworth A.S., Howson J.M.M., Assimes T.L., Chowdhury R., Orho-Melander M., Damrauer S. (2017). Identification of new susceptibility loci for type 2 diabetes and shared etiological pathways with coronary heart disease. Nat. Genet..

[46] Li H., Wetten S., Li L., Jean P.L.S., Upmanyu R., Surh L., Hosford D., Barnes M.R., Briley J.D., Borrie M. (2008). Candidate Single-Nucleotide Polymorphisms from a Genomewide Association Study of Alzheimer Disease. Arch. Neurol..

[47] Webster J.A., Myers A.J., Pearson J.V., Craig D.W., Hu-Lince D., Coon K.D., Zismann V.L., Beach T., Leung D., Bryden L. (2007). Sorl1 as an Alzheimer’s Disease Predisposition Gene?. Neurodegener. Dis..

[48] The AMD Gene Consortium (2013). Seven new loci associated with age-related macular degeneration. Nat. Genet..

[49] Van der Harst P., Verweij N. (2018). Identification of 64 Novel Genetic Loci Provides an Expanded View on the Genetic Architecture of Coronary Artery Disease. Circ. Res..

[50] Joshi P.K., Pirastu N., Kentistou K.A., Fischer K., Hofer E., Schraut K.E., Clark D.W., Nutile T., Barnes C.L.K., Timmers P.R.H.J. (2017). Genome-wide meta-analysis associates HLA-DQA1/DRB1 and LPA and lifestyle factors with human longevity. Nat. Commun..

[51] Pilling L.C., Kuo C.-L., Sicinski K., Tamosauskaite J., Kuchel G., Harries L.W., Herd P., Wallace R., Ferrucci L., Melzer D. (2017). Human longevity: 25 genetic loci associated in 389,166 UK biobank participants. Aging.

[52] Spracklen C.N., Chen P., Kim Y.J., Wang X., Cai H., Li S., Long J., Wu Y., Wang Y.X., Takeuchi F. (2017). Association analyses of East Asian individuals and trans-ancestry analyses with European individuals reveal new loci associated with cholesterol and triglyceride levels. Hum. Mol. Genet..

[53] Wojcik G.L., Graff M., Nishimura K.K., Tao R., Haessler J., Gignoux C.R., Highland H.M., Patel Y.M., Sorokin E.P., Avery C.L. (2019). Genetic analyses of diverse populations improves discovery for complex traits. Nature.

[54] Klarin D., Damrauer S.M., Cho K., Sun Y.V., Teslovich T.M., Honerlaw J., Gagnon D.R., Duvall S.L., Li J., Peloso G.M. (2018). Genetics of blood lipids among ~300,000 multi-ethnic participants of the Million Veteran Program. Nat. Genet..

[55] De Vries P.S., Brown M.R., Bentley A.R., Sung Y.J., Winkler T.W., Ntalla I., Schwander K., Kraja A.T., Guo X., Franceschini N. (2019). Multiancestry Genome-Wide Association Study of Lipid Levels Incorporating Gene-Alcohol Interactions. Am. J. Epidemiology.

[56] Moore J.H. (2005). A global view of epistasis. Nat. Genet..

[57] Moore J.H., Gilbert J.C., Tsai C.-T., Chiang F.-T., Holden T., Barney N., White B.C. (2006). A flexible computational framework for detecting, characterizing, and interpreting statistical patterns of epistasis in genetic studies of human disease susceptibility. J. Theor. Biol..

[58] Paththinige C., Sirisena N., Dissanayake V. (2017). Genetic determinants of inherited susceptibility to hypercholesterolemia—A comprehensive literature review. Lipids Heal. Dis..

[59] Jong M.C., Hofker M.H., Havekes L.M. (1999). Role of ApoCs in Lipoprotein Metabolism. Arter. Thromb. Vasc. Biol..

[60] Xie G., Myint P.K., Voora D., Laskowitz D.T., Shi P., Ren F., Wang H., Yang Y., Huo Y., Gao W. (2015). Genome-wide association study on progression of carotid artery intima media thickness over 10 years in a Chinese cohort. Atherosclerosis.

[61] De Vries R., Dallinga-Thie G.M., Smit A.J., Wolffenbuttel B.H.R., van Tol A., Dullaart R.P.F. (2005). Elevated plasma phospholipid transfer protein activity is a determinant of carotid intima-media thickness in type 2 diabetes mellitus. Diabetologia.

[62] Abdushi S.A., Nazreku F.D., Kryeziu F.U. (2013). Increased carotid intima-media thickness associated with high hs-CRP levels is a predictor of unstable coronary artery disease: Cardiovascular topic. Cardiovasc. J. Afr..

[63] Paraskevas K.I. (2015). Increased carotid intima-media thickness and coronary artery disease share more links besides inflammation. Int. J. Cardiol..

[64] Ligthart S., Vaez A., Hsu Y.-H., Stolk R., Uitterlinden A.G., Hofman A., Alizadeh B.Z., Franco O., Dehghan A. (2016). Bivariate genome-wide association study identifies novel pleiotropic loci for lipids and inflammation. BMC Genom..

[65] Germain M., Chasman D.I., De Haan H., Tang W., Lindström S., Weng L.-C., De Andrade M., De Visser M.C., Wiggins K.L., Suchon P. (2015). Meta-analysis of 65,734 Individuals Identifies TSPAN15 and SLC44A2 as Two Susceptibility Loci for Venous Thromboembolism. Am. J. Hum. Genet..

[66] Hinds D.A., Buil A., Ziemek D., Martinez-Perez A., Malik R., Folkersen L., Germain M., Mälarstig A., Brown A., Soria J.M. (2016). Genome-wide association analysis of self-reported events in 6135 individuals and 252 827 controls identifies 8 loci associated with thrombosis. Hum. Mol. Genet..

[67] Nordestgaard B.G., Chapman M.J., Ray K., Borén J., Andreotti F., Watts G., Ginsberg H., Amarenco P., Catapano A.L., Descamps O.S. (2010). Lipoprotein(a) as a cardiovascular risk factor: Current status. Eur. Hear. J..

[68] Yang X., Sethi A., Yanek L.R., Knapper C., Nordestgaard B.G., Tybjærg-Hansen A., Becker D.M., Mathias R.A., Remaley A.T., Becker L.C. (2016). SCARB1 Gene Variants Are Associated with the Phenotype of Combined High High-Density Lipoprotein Cholesterol and High Lipoprotein (a). Circ. Cardiovasc. Genet..

[69] Hebbar P., Nizam R., Melhem M., Alkayal F., Elkum N., John S.E., Tuomilehto J., Alsmadi O., Thanaraj T.A. (2018). Genome-wide association study identifies novel recessive genetic variants for high TGs in an Arab population. J. Lipid Res..

[70] Borthwick F., Allen A.-M., Taylor J.M., Graham A. (2010). Overexpression of STARD3 in human monocyte/macrophages induces an anti-atherogenic lipid phenotype. Clin. Sci..

[71] Winkler T.W., Justice A.E., Graff M., Barata L., Feitosa M.F., Chu S., Czajkowski J., Esko T., Fall T., Kilpeläinen T.O. (2015). The Influence of Age and Sex on Genetic Associations with Adult Body Size and Shape: A Large-Scale Genome-Wide Interaction Study. PLoS Genet..

[72] Shungin D., Winkler T.W., Croteau-Chonka D.C., Ferreira T., Locke A.E., Mägi R., Strawbridge R.J., Pers T.H., Fischer K., Justice A.E. (2015). New genetic loci link adipose and insulin biology to body fat distribution. Nature.

[73] Rockman M.V., Kruglyak L. (2006). Genetics of global gene expression. Nat. Rev. Genet..

[74] Claudel T., Inoue Y., Barbier O., Duran-Sandoval D., Kosykh V., Fruchart J., Fruchart J.-C., Gonzalez F.J., Staels B. (2003). Farnesoid X receptor agonists suppress hepatic apolipoprotein CIII expression. Gastroenterology.

[75] Jakobsson T., Venteclef N., Toresson G., Damdimopoulos A.E., Ehrlund A., Lou X., Sanyal S., Steffensen K.R., Gustafsson J., Treuter E. (2009). GPS2 Is Required for Cholesterol Efflux by Triggering Histone Demethylation, LXR Recruitment, and Coregulator Assembly at the ABCG1 Locus. Mol. Cell.

[76] Gadaleta R.M., Van Erpecum K.J., Oldenburg B., Willemsen E.C.L., Renooij W., Murzilli S., Klomp L.W.J., Siersema P.D., Schipper M.E., Danese S. (2011). Farnesoid X receptor activation inhibits inflammation and preserves the intestinal barrier in inflammatory bowel disease. Gut.

[77] Schulte K.W., Green E., Wilz A., Platten M., Daumke O. (2017). Structural Basis for Aryl Hydrocarbon Receptor-Mediated Gene Activation. Structure.

[78] Seok S.-H., Lee W., Jiang L., Molugu K., Zheng A., Li Y., Park S., Bradfield C.A., Xing Y. (2017). Structural hierarchy controlling dimerization and target DNA recognition in the AHR transcriptional complex. Proc. Natl. Acad. Sci. USA.

[79] Alshaarawy O., Elbaz H., Andrew M.E. (2016). The association of urinary polycyclic aromatic hydrocarbon biomarkers and cardiovascular disease in the US population. Environ. Int..

[80] Amin M.-M., Poursafa P., Moosazadeh M., Abedini E., Hajizadeh Y., Mansourian M., Pourzamani H. (2017). A systematic review on the effects of polycyclic aromatic hydrocarbons on cardiometabolic impairment. Int. J. Prev. Med..

[81] Alhamdow A., Lindh C., Albin M., Gustavsson P., Tinnerberg H., Broberg K. (2017). Early markers of cardiovascular disease are associated with occupational exposure to polycyclic aromatic hydrocarbons. Sci. Rep..

[82] Vogel C.F.A., Sciullo E., Matsumura F. (2004). Activation of Inflammatory Mediators and Potential Role of Ah-Receptor Ligands in Foam Cell Formation. Cardiovasc. Toxicol..

[83] Liu S., Cai X., Wu J., Cong Q., Chen X., Li T., Du F., Ren J., Wu Y.-T., Grishin N.V. (2015). Phosphorylation of innate immune adaptor proteins MAVS, STING, and TRIF induces IRF3 activation. Science.

[84] Joehanes R., Ying S., Huan T., Johnson A.D., Raghavachari N., Wang R., Liu P., Woodhouse K.A., Sen S.K., Tanriverdi K. (2013). Gene Expression Signatures of Coronary Heart Disease. Arter. Thromb. Vasc. Biol..

[85] Mishiro T., Ishihara K., Hino S., Tsutsumi S., Aburatani H., Shirahige K., Kinoshita Y., Nakao M. (2009). Architectural roles of multiple chromatin insulators at the human apolipoprotein gene cluster. EMBO J..

[86] Cayrol C., Lacroix C., Mathe C., Ecochard V., Ceribelli M., Loreau E., Lazar V., Dessen P., Mantovani R., Aguilar L. (2006). The THAP–zinc finger protein THAP1 regulates endothelial cell proliferation through modulation of pRB/E2F cell-cycle target genes. Blood.

[87] McPherson A., Larson S.B. (2019). The structure of human apolipoprotein C-1 in four different crystal forms. J. Lipid Res..

[88] Lowe J.K., Maller J.B., Pe’Er I., Neale B.M., Salit J., Kenny E.E., Shea J.L., Burkhardt R., Smith J.G., Ji W. (2009). Genome-Wide Association Studies in an Isolated Founder Population from the Pacific Island of Kosrae. PLoS Genet..

[89] Deelen J., Beekman M., Uh H.-W., Broer L., Ayers K.L., Tan Q., Kamatani Y., Bennet A.M., Tamm R., Trompet S. (2014). Genome-wide association meta-analysis of human longevity identifies a novel locus conferring survival beyond 90 years of age. Hum. Mol. Genet..

[90] Cheng C., Tempel D., Dekker W.K.D., Haasdijk R., Chrifi I., Bos F.L., Wagtmans K., Van De Kamp E.H., Blonden L., Biessen E.A. (2011). Ets2 Determines the Inflammatory State of Endothelial Cells in Advanced Atherosclerotic Lesions. Circ. Res..

[91] Glenn K.C., Frost G.H., Bergmann J.S., Carney D.H. (1988). Synthetic peptides bind to high-affinity thrombin receptors and modulate thrombin mitogenesis. Pept. Res..

[92] Borissoff J.I., Spronk H.M., Heeneman S., Cate H.T. (2009). Is thrombin a key player in the ’coagulation-atherogenesis’ maze?. Cardiovasc. Res..

[93] Borissoff J.I., Spronk H.M., Cate H.T. (2011). The Hemostatic System as a Modulator of Atherosclerosis. N. Engl. J. Med..

[94] Zhu X., Li J., Deng S., Yu K., Liu X., Deng Q., Sun H., Zhang X., He M., Guo H. (2016). Genome-Wide Analysis of DNA Methylation and Cigarette Smoking in a Chinese Population. Environ. Health Perspect..

[95] Goodale B.C., Tilton S.C., Corvi M.M., Wilson G.R., Janszen D.B., Anderson K.A., Waters K., Tanguay R.L. (2013). Structurally distinct polycyclic aromatic hydrocarbons induce differential transcriptional responses in developing zebrafish. Toxicol. Appl. Pharmacol..

[96] Jorgensen E.M., Alderman M.H., Taylor H.S. (2016). Preferential epigenetic programming of estrogen response after in utero xenoestrogen (bisphenol-A) exposure. FASEB J..

[97] Collí-Dulá R.C., Friedman M.A., Hansen B., Denslow N.D. (2016). Transcriptomics analysis and hormonal changes of male and female neonatal rats treated chronically with a low dose of acrylamide in their drinking water. Toxicol. Rep..

[98] Gai Z., Hiller C., Chin S.H., Hofstetter L., Stieger B., Konrad D., Kullak-Ublick G.A. (2014). Uninephrectomy augments the effects of high fat diet induced obesity on gene expression in mouse kidney. Biochim. Biophys. Acta (BBA) Mol. Basis Dis..

[99] Sui Y., Park S.-H., Wang F., Zhou C. (2018). Perinatal Bisphenol A Exposure Increases Atherosclerosis in Adult Male PXR-Humanized Mice. Endocrinology.

[100] Yordy J.S., Moussa O., Pei H., Chaussabel D., Li R., Watson D.K. (2004). SP100 inhibits ETS1 activity in primary endothelial cells. Oncogene.

[101] Brasch J., Harrison O.J., Ahlsen G., Carnally S.M., Henderson R.M., Honig B., Shapiro L. (2011). Structure and Binding Mechanism of Vascular Endothelial Cadherin: A Divergent Classical Cadherin. J. Mol. Biol..

[102] Verschuur M., de Jong M., Felida L., de Maat M.P., Vos H.L. (2005). A Hepatocyte Nuclear Factor-3 Site in the Fibrinogen β Promoter Is Important for Interleukin 6-induced Expression, and Its Activity Is Influenced by the Adjacent −148C/T Polymorphism. J. Biol. Chem..

[103] The International HapMap 3 Consortium (2010). Integrating common and rare genetic variation in diverse human populations. Nature.

[104] Tiret L., Poirier O., Nicaud V., Barbaux S., Herrmann S.M., Perret C., Raoux S., Francomme C., Lebard G., Trégouët D. (2002). Heterogeneity of linkage disequilibrium in human genes has implications for association studies of common diseases. Hum. Mol. Genet..

[105] Azarova I., Klyosova E., Polonikov A. (2021). The Link between Type 2 Diabetes Mellitus and the Polymorphisms of Glutathione-Metabolizing Genes Suggests a New Hypothesis Explaining Disease Initiation and Progression. Life.

