# Supplementary material for: Comprehensive Statistical and Bioinformatics Analysis in the Deciphering of Putative Mechanisms by Which Lipid-Associated GWAS Loci Contribute to Coronary Artery Disease
Source: Biomedicines. 2022 Jan 25;10(2):259. doi: 10.3390/biomedicines10020259 (PMC8868589; doi:10.3390/biomedicines10020259)
Supplement: Supplementary file 1 [file biomedicines-10-00259-s001.zip › Supplementary table S1.pdf]

Supplementary Table S1. Binding sites of transcription factors predicted at the studied gene polymorphisms

Gene (SNP ID)	Allele	Transcription factors whose binding sites were predicted in the presence of particular allele at the studied SNPs[1]			
		Activators	Repressors	Activators/Repressors	unknown effect
ABCA1 (rs1883025)	C	AIRE, AP1, BDP1, E2F3, EBF1, ETS, HNF4, NFAT, NFIC, NR2C2, PAX5, REST, PRDM1, RUNX3, RXRA, SP1, SPI1	-	ATF3, YY1, HAND1, Hand1::Tcfe2a, SIN3A	CACBP, TATA
	T	PITX2, HNF4A, NHLH1, PITX1	-	SIN3A, PAX, PPARG, ZKSCAN3	OBOX1
APOC1 (rs4420638)	A	HMBOX1	-		-
	G	EGR1, ETS1, ETS2, FIGLA, FLI1, NFE2L2, SPDEF, TCF12, TCF4, ZIC1, E2F1	ZBTB18, FEV	SRF, SIN3A, ZEB1, SOX2, ELF3, GLI, SP1, EHF, TCF3, ELF1, ELK4	-
CETP (rs3764261)	C	GCM1, GCM2, MTF1, TBX5, TFAP2, ZBTB33, ZIC3, ZNF143, ZNF410	-	GLI2, TOPORS	-
	A	TFCP2, FOXJ1, LEF1, MYF, NFY, NFYA, RAD21, RUNX2, SIX5, SRY	FOXK1	ZEB1, FOXL1	-
COBLL1 (rs12328675)	T	POU1F1, RUNX1, SOX8, TBP	MYEF2, NFIL3	FOXA2, GATA3	
	C	AP1, JUN, POU2F1, RARB, RBPJ, SMAD2::SMAD3::SMAD4	-	E2F8, TFAP2C, BACH2	SOAT1
F2 (rs3136441)	T	AR, FOXA1, FOXF1, FOXI1, FOXJ2, RXRA, SOX17, SRY	FOXG1	SOX2, SOX7, FOXA2	-
	C	E2F2, ETS, HNF4A	BHLHE23, CBX5	TCF21	ZFP423
GALNT2 (rs4846914)	A	E2F, HLTF, REST	E2F1, SCRT1, SCRT2	PPARA	-
	G	PAX5, EBF1, ESRRA, NKX2-1, ZNF143	-	-	-

LILRA3 (rs386000)	G	-	-	-	-
	C	-	-	-	-
LPA (rs55730499)	C	AR, E2F2, E2F3, ESR1, GCM2, NR3C1	CTCF, HIC2,	MLX, THAP1	-
	T	NR1H, AHR::ARNT, IRF	HAND1	-	SMAD
NPC1L1 (rs217406)	C	NFIX, NFY, RAD21, RORA, TBXT	-	PKNOX2, SIX4	HERPUD1, ZNF784
	G	AR, ATF1, CREB3L1, CREB3L2, GMEB1, TEF, XBP1	-	-	-
PLTP (rs6065906)	T	HOXD13, AP1, CDX1, CDX2, E2F, GBX1, GBX2, HMX2, HMX3, HOXA10, HOXC10, HOXD8, IRF, ISX, LMX1B, MEF2A, NFY, POU6F2, RAX, RAX2, SHOX, SOX9, SP1, SRY	SOX21, EN1, MSX1, MSX2, PAX4, POU5F1, Pou5f1::Sox2, PRRX1	ALX1, CPEB1, MZF1, NKX6-1, Pou5f1::Sox2	ALX3, GSX2, LBX2, SHOX2, VSX1
	C	BRCA1, FOXO1, RUNX1	-	-	-
PSKH1 (rs16942887)	G	RUNX2, HNF4A, RAD21, RUNX1, RUNX2, RUNX3, SPIB, STAT4	GFI1B	Hand1::Tcfe2a, ZNF354C	RUNX
	A	HOXA4, HOXA5, PRDM1	TBX15	-	-
ST3GAL4 (rs11220463)	A	ARNT, ATF4, CREB, CREB3L1, CREB3L2, HIF1A, HIF1A::ARNT, MYCN, USF1, XBP1	E2F1, MLX, THAP1, CLOCK::ARNTL, ZBTB7A	MAX, YY1, MYC::MAX, YY2	-
	T	ELF1, HOXC13, IRF, MEIS1::HOXA9, MYF, RAD21, RARB, SP1	-	-	HOXA11
STARD3 (rs881844)	G	BDP1, E2F8, ETS, ETS1, MTF1, NFE2L2, SIX5, HLTF, ZNF143	-	EHF, ELK3	-
	C	CLOCK::ARNTL, AHR, EGR, EGR1, FOXO3, HEY2, KLF4, MTF1, MYCN, NHLH1, NKX2-1, NRF1, SP1, USF, VDR	PAX4, FOXG1, MXI1, MYC	ZEB1, SRF, PPARG, LMO2	NKX2-5, PTEN, TATA
ZNF648	A	RUNX1, E2F, IRF, PBX1, PBX3, TEAD1	GFI1	-	-

(rs1689800)	G	BRCA1, FOXO1, GMEB2, NFY, NR2C2, RAD21, RARG, REST, RFX4, RXRA::VDR, SP1, VDR, ZIC3, ZNF410	ZIC1	NR2F1, PPARA	RFX7, SCAP, ZNF740, ZNF784
SCARB1 (rs838880)	T	FOXA, FOXI1, FOXJ1, FOXO1, FOXO4, NFIC, RFX5, SMAD3, SOX8, SOX9	E2F1, SOX21, PAX4, HIC1, HIC2, FOXQ1	ZEB1, HAND1, SOX2, FOXA2	COMP1, DMRTA1
	C	SPDEF	ERF	NR1H4	-
PPP1R3B (rs9987289)	G	ATF1, ATF2, E2F2, E2F3, E2F4, EGR1, GMEB1, MYBL2, PAX5, SPDEF, TBXT, TBX1, TBX4, ZBTB33	E2F1, TBX2, TBX21, ZBTB7B	TBR1, NR2F1, MGA, EOMES, CREB1, SRF, PPARG, TBX20	-
	A	CUX1, ARID3A	HIC2, MAFF	PPARG, MAFB	AFP, PAX7

[1] TFBS were in silico predicted by the atSNP tool (http://atsnp.biostat.wisc.edu)
