# Supplementary material for: Comprehensive Statistical and Bioinformatics Analysis in the Deciphering of Putative Mechanisms by Which Lipid-Associated GWAS Loci Contribute to Coronary Artery Disease
Source: Biomedicines. 2022 Jan 25;10(2):259. doi: 10.3390/biomedicines10020259 (PMC8868589; doi:10.3390/biomedicines10020259)
Supplement: Supplementary file 1 [file biomedicines-10-00259-s001.zip › Supplementary table S2.pdf]

Supplementary Table S2. ***Trans*-QTLs for eight lipid-associated GWAS polymorphisms**

SNP Id	Gene Symbol	P-Value	NES	Tissue
rs3136441				
rs3136441	*ACP2*	6.7E-10	-0.37	Esophagus - Mucosa
rs3136441	*ACP2*	9.8E-08	-0.27	Skin - Sun Exposed (Lower leg)
rs3136441	*ACP2*	2.7E-07	-0.29	Skin - Not Sun Exposed (Suprapubic)
rs3136441	*ACP2*	5E-07	-0.2	Muscle - Skeletal
rs3136441	*ACP2*	7.3E-07	-0.22	Thyroid
rs3136441	*ACP2*	8.8E-06	-0.44	Spleen
rs3136441	*AGBL2*	0.00014	0.3	Artery - Tibial
rs3136441	*ARHGAP1*	2.1E-08	-0.21	Skin - Sun Exposed (Lower leg)
rs3136441	*ARHGAP1*	2.8E-08	-0.22	Muscle - Skeletal
rs3136441	*ARHGAP1*	5.5E-07	-0.22	Nerve - Tibial
rs3136441	*ARHGAP1*	1.1E-06	-0.37	Pancreas
rs3136441	*ARHGAP1*	5.2E-06	-0.21	Skin - Not Sun Exposed (Suprapubic)
rs3136441	*ARHGAP1*	9.9E-06	-0.28	Breast - Mammary Tissue
rs3136441	*ARHGAP1*	0.000016	-0.19	Adipose - Subcutaneous
rs3136441	*ARHGAP1*	0.000019	-0.2	Esophagus - Mucosa
rs3136441	*ARHGAP1*	0.000033	-0.18	Thyroid
rs3136441	*ARHGAP1*	0.000042	-0.17	Lung
rs3136441	*ARHGAP1*	0.000043	-0.39	Pituitary
rs3136441	*ARHGAP1*	0.000046	-0.26	Stomach
rs3136441	*ARHGAP1*	0.000091	-0.23	Adipose - Visceral (Omentum)
rs3136441	*C11orf49*	8.7E-07	0.28	Nerve - Tibial
rs3136441	*DDB2*	2E-07	0.22	Skin - Sun Exposed (Lower leg)
rs3136441	*LRP4*	8.8E-10	-0.58	Pancreas
rs3136441	*LRP4*	2E-09	0.4	Nerve - Tibial
rs3136441	*LRP4-AS1*	2.1E-07	-0.57	Pancreas
rs3136441	*LRP4-AS1*	2.6E-06	0.36	Nerve - Tibial
rs3136441	*NR1H3*	0.00014	-0.2	Testis
rs3136441	*RAPSN*	0.000071	0.34	Skin - Not Sun Exposed (Suprapubic)
rs3136441	*ZNF408*	6.3E-09	-0.32	Artery - Tibial
rs3136441	*ZNF408*	2.1E-07	-0.35	Artery - Aorta
rs3136441	*ZNF408*	2.5E-06	-0.27	Nerve - Tibial
rs3136441	*ZNF408*	0.000014	-0.23	Thyroid
rs3136441	*ZNF408*	0.000055	-0.32	Breast - Mammary Tissue
rs881844				
rs881844	*AC087491.2*	3.1E-07	0.32	Skin - Not Sun Exposed (Suprapubic)
rs881844	*AC087491.2*	1.4E-06	0.26	Skin - Sun Exposed (Lower leg)
rs881844	*ERBB2*	3.9E-07	-0.17	Nerve - Tibial
rs881844	*ERBB2*	0.000014	-0.15	Heart - Atrial Appendage
rs881844	*ERBB2*	0.00003	-0.081	Skin - Sun Exposed (Lower leg)
rs881844	*GSDMA*	0.000053	0.28	Adipose - Subcutaneous
rs881844	*GSDMB*	0.000013	-0.13	Whole Blood
rs881844	*GSDMB*	0.000021	-0.11	Stomach
rs881844	*GSDMB*	0.000025	-0.32	Spleen

rs881844	*ORMDL3*	4.6E-06	-0.48	Cells - EBV-transformed lymphocytes
rs881844	*ORMDL3*	0.000034	-0.16	Whole Blood
rs881844	*PGAP3*	3E-17	-0.27	Thyroid
rs881844	*PGAP3*	3.5E-15	-0.34	Heart - Left Ventricle
rs881844	*PGAP3*	4.1E-14	-0.26	Stomach
rs881844	*PGAP3*	9.6E-14	-0.26	Nerve - Tibial
rs881844	*PGAP3*	1.6E-13	-0.55	Pituitary
rs881844	*PGAP3*	3.5E-13	-0.19	Colon - Transverse
rs881844	*PGAP3*	5.6E-13	-0.19	Muscle - Skeletal
rs881844	*PGAP3*	1.9E-10	-0.19	Skin - Sun Exposed (Lower leg)
rs881844	*PGAP3*	2.2E-10	-0.28	Heart - Atrial Appendage
rs881844	*PGAP3*	5.2E-10	-0.17	Lung
rs881844	*PGAP3*	1.2E-08	-0.17	Breast - Mammary Tissue
rs881844	*PGAP3*	3.1E-08	-0.29	Pancreas
rs881844	*PGAP3*	3.6E-08	-0.23	Adipose - Visceral (Omentum)
rs881844	*PGAP3*	6.9E-08	-0.17	Esophagus - Muscularis
rs881844	*PGAP3*	3.3E-07	-0.31	Adrenal Gland
rs881844	*PGAP3*	4.1E-07	-0.24	Liver
rs881844	*PGAP3*	0.00001	-0.14	Skin - Not Sun Exposed (Suprapubic)
rs881844	*PGAP3*	0.000018	-0.19	Artery - Aorta
rs881844	*PNMT*	7.6E-13	-0.27	Heart - Atrial Appendage
rs881844	*PNMT*	6E-07	-0.17	Muscle - Skeletal
rs881844	*PNMT*	0.000036	-0.11	Lung
rs881844	*PNMT*	0.000052	-0.11	Nerve - Tibial
rs881844	*PPP1R1B*	1.1E-14	0.36	Esophagus - Mucosa
rs881844	*PPP1R1B*	3.9E-08	-0.38	Heart - Atrial Appendage
rs881844	*PPP1R1B*	4.3E-06	0.12	Skin - Not Sun Exposed (Suprapubic)
rs881844	*PPP1R1B*	7.9E-06	0.088	Skin - Sun Exposed (Lower leg)
rs881844	*RP11-690G19.3*	5.4E-07	0.17	Thyroid
rs16942887				
rs16942887	*ACD*	0.00015	-0.14	Adipose - Subcutaneous
rs16942887	*CTRL*	7.1E-08	0.35	Skin - Sun Exposed (Lower leg)
rs16942887	*CTRL*	0.000067	0.15	Whole Blood
rs16942887	*CTRL*	0.00012	0.3	Skin - Not Sun Exposed (Suprapubic)
rs16942887	*DPEP3*	5.1E-07	-0.2	Whole Blood
rs16942887	*DUS2*	0.000006	0.2	Whole Blood
rs16942887	*DUS2*	0.000058	0.14	Cells - Transformed fibroblasts
rs16942887	*GFOD2*	3.3E-12	-0.23	Esophagus - Mucosa
rs16942887	*GFOD2*	2.3E-07	-0.21	Skin - Sun Exposed (Lower leg)
rs16942887	*LCAT*	3E-10	0.27	Nerve - Tibial
rs16942887	*LCAT*	4.2E-09	0.25	Whole Blood
rs16942887	*LCAT*	5.6E-09	0.24	Adipose - Subcutaneous
rs16942887	*LCAT*	0.000001	0.17	Cells - Transformed fibroblasts
rs16942887	*LCAT*	1.1E-06	0.18	Adipose - Visceral (Omentum)
rs16942887	*LCAT*	1.9E-06	0.23	Testis
rs16942887	*NFATC3*	4.3E-06	0.25	Testis

rs16942887	*PRMT7*	1E-10	0.39	Esophagus - Mucosa
rs16942887	*PRMT7*	4.2E-09	0.37	Thyroid
rs16942887	*PRMT7*	4.3E-09	0.31	Heart - Left Ventricle
rs16942887	*PRMT7*	1.2E-08	0.31	Artery - Tibial
rs16942887	*PRMT7*	1.2E-07	0.26	Adipose - Visceral (Omentum)
rs16942887	*PRMT7*	2.3E-07	0.2	Muscle - Skeletal
rs16942887	*PRMT7*	4.5E-07	0.26	Cells - Transformed fibroblasts
rs16942887	*PRMT7*	8.3E-07	0.51	Adrenal Gland
rs16942887	*PRMT7*	9.3E-07	0.33	Esophagus - Muscularis
rs16942887	*PRMT7*	1.1E-06	0.21	Lung
rs16942887	*PRMT7*	8.6E-06	0.35	Esophagus - Gastroesophageal Junction
rs16942887	*PRMT7*	0.00001	0.37	Colon - Transverse
rs16942887	*PRMT7*	0.000016	0.26	Nerve - Tibial
rs16942887	*PRMT7*	0.000017	0.41	Artery - Coronary
rs16942887	*PRMT7*	0.000019	0.23	Skin - Sun Exposed (Lower leg)
rs16942887	*PRMT7*	0.000022	0.24	Adipose - Subcutaneous
rs16942887	*PRMT7*	0.000028	0.32	Artery - Aorta
rs16942887	*PRMT7*	0.000032	0.33	Pituitary
rs16942887	*PRMT7*	0.000039	0.13	Whole Blood
rs16942887	*PRMT7*	0.000045	0.23	Heart - Atrial Appendage
rs16942887	*PSMB10*	0.000026	-0.33	Testis
rs16942887	*SLC12A4*	1.8E-06	0.17	Cells - Transformed fibroblasts
rs16942887	*SLC12A4*	6.3E-06	0.21	Adipose - Subcutaneous
rs16942887	*SLC12A4*	9.1E-06	0.17	Whole Blood
rs16942887	*TSNAXIP1*	2.5E-10	0.28	Thyroid
rs16942887	*TSNAXIP1*	1.2E-06	0.28	Esophagus - Muscularis
rs16942887	*TSNAXIP1*	0.000023	0.33	Adrenal Gland
rs16942887	*TSNAXIP1*	0.000062	0.41	Brain - Cerebellar Hemisphere
rs16942887	*TSNAXIP1*	0.00014	0.22	Esophagus - Mucosa
rs217406				
rs217406	*NUDCD3*	1.5E-18	0.51	Thyroid
rs217406	*NUDCD3*	1.3E-11	0.31	Esophagus - Muscularis
rs217406	*NUDCD3*	2.1E-09	0.38	Esophagus - Gastroesophageal Junction
rs217406	*NUDCD3*	8.1E-09	0.31	Nerve - Tibial
rs217406	*NUDCD3*	3.3E-08	0.19	Lung
rs217406	*NUDCD3*	7.8E-08	0.2	Artery - Tibial
rs217406	*NUDCD3*	5.8E-07	0.18	Skin - Not Sun Exposed (Suprapubic)
rs217406	*NUDCD3*	7.5E-07	0.23	Adipose - Subcutaneous
rs217406	*NUDCD3*	9E-07	0.18	Whole Blood
rs217406	*NUDCD3*	2.2E-06	0.3	Colon - Sigmoid
rs217406	*NUDCD3*	2.8E-06	0.23	Artery - Aorta
rs217406	*NUDCD3*	4.7E-06	0.37	Brain - Cerebellum
rs217406	*NUDCD3*	4.7E-06	0.2	Esophagus - Mucosa
rs217406	*NUDCD3*	1.1E-05	0.5	Spleen
rs217406	*NUDCD3*	1.7E-05	0.31	Breast - Mammary Tissue
rs217406	*NUDCD3*	4.2E-05	0.16	Muscle - Skeletal

rs217406	*NUDCD3*	5.6E-05	0.13	Skin - Sun Exposed (Lower leg)
rs6065906				
rs6065906	*NEURL2*	1.8E-11	-0.46	Adipose - Visceral (Omentum)
rs6065906	*NEURL2*	0.000016	-0.27	Adipose - Subcutaneous
rs6065906	*RP3-337O18.9*	0.000018	-0.29	Adipose - Visceral (Omentum)
rs6065906	*SPATA25*	0.000023	-0.19	Testis
rs6065906	*SNX21*	0.000026	0.16	Muscle - Skeletal
rs6065906	*SNX21*	0.000037	-0.2	Thyroid
rs6065906	*RP3-337O18.9*	0.000049	-0.24	Adipose - Subcutaneous
rs3764261				
rs3764261	*NLRC5*	1.4e-13	-0.32	Cells - Transformed fibroblasts
rs12328675				
rs12328675	*GRB14*	7.4e-13	-0.58	Esophagus - Muscularis
rs55730499				
rs55730499	*SLC22A3*	4.7e-7	0.49	Skin - Sun Exposed (Lower leg)
